# Burden of carbapenem non-susceptible infections in high-risk patients: systematic literature review and meta-analysis

**DOI:** 10.1186/s13756-020-00858-8

**Published:** 2020-12-07

**Authors:** Esther E. Avendano, Gowri Raman, Jeffrey Chan, Eilish McCann

**Affiliations:** 1 Institute for Clinical Research and Health Policy Studies, Center for Clinical Evidence Synthesis, Tufts Medical Center, Boston, MA USA; 2 Tufts University School of Medicine, Boston, MA USA; 3 Merck & Co., Inc., Kenilworth, NJ USA

**Keywords:** Carbapenem-resistance, Gram-negative, High-risk, Mortality

## Abstract

**Background:**

Owing to their resistance to an important class of antibiotics, the prevention and treatment of carbapenem-resistant (CR)/non-susceptible Gram-negative (GN) infections has become an important public health objective. We conducted a systematic review and meta-analysis of published literature to evaluate the burden of CR GN infections, focusing on high-risk patients such as transplant recipients, or patients with cancer, renal impairment, or sepsis.

**Methods:**

MEDLINE®, Cochrane Central, and Embase® were searched between 2010 and March 2019. Abstracts and full-text articles were screened in duplicate. Random effects meta-analysis was conducted when reported outcomes were sufficiently similar.

**Results:**

Twenty-six publications were eligible. Meta-analyses found increased mortality associated with CR infections among high-risk patients in both unadjusted analysis (8 studies; summary unadjusted odds ratio [OR]: 5.85; 95% confidence interval [CI]: 3.69, 9.26; I^2^ = 19.8%) and adjusted analysis (5 studies; summary hazard ratio [HR]: 4.67; 95% CI: 2.18, 9.99; I^2^ = 77.7%), compared to patients with carbapenem-susceptible (CS) infections or no infection. Increased mortality was also seen in subgroup analyses by length of follow-up (either short-term or long-term) or causative pathogen. A limited number of studies found that CR GN infections increased the risk for mechanical ventilation, adverse events such as graft failure or acute rejection in solid organ transplant recipients, increased renal failure or nephrotoxicity, and an increase in readmissions and costs, though the findings reported in the literature were not consistent.

**Conclusion:**

This systematic literature review and meta-analysis indicates that CR GN infections in high-risk patients are associated with increased mortality, emphasizing the need for antimicrobial stewardship and infection control in hospitals which treat high-risk patients and for the development of effective antimicrobials with favorable efficacy and safety profiles for the treatment of CR GN infections.

## Introduction

An alarming increase in antibiotic-resistant Gram-negative (GN) infections represents a burden on healthcare systems globally [1–3]. The Centers for Disease Control classifies carbapenem-resistant (CR) Enterobacteriaceae as a major public health threat and the World Health Organization considers CR *Pseudomonas aeruginosa* as a critical priority pathogen requiring immediate and aggressive action [4]. CR/carbapenem non-susceptible GN pathogens are typically resistant to most of the antibiotics used in routine clinical practice, in addition to the carbapenem class. The increase in CR GN infections has led to a resurgence in the use of older antibiotics i.e., broad antimicrobial therapies such as polymyxins that were seldom utilized in the recent past due to efficacy, dosing, and/or toxicity concerns [5]. As a result of these factors, the prevention and effective treatment of CR GN infections has become an important public health objective.

Certain risk factors increase the likelihood of a patient becoming colonized or infected with GN bacteria. These include exposure to healthcare settings [6–9], use of invasive devices or procedures [10–12], receipt of prior antimicrobial therapy [13], foreign travel [14, 15], and being classified as ‘high-risk’ [16–18]. To expand upon the latter, ‘high-risk’ can refer to certain clinical characteristics or comorbidities (e.g., hematological or solid malignancies), receipt of concomitant medication(s), or surgical procedures (e.g., organ transplant). In addition to having a greater risk of acquiring GN pathogens, organ transplant recipients, patients undergoing major surgeries, and immunosuppressed patients are predisposed to poor clinical outcomes including increased morbidity and mortality; this is further compounded by the GN infection [19, 20]. No recent literature review has examined the burden of CR GN infections (and the burden relative to that of carbapenem-susceptible [CS] GN infections) among these high-risk patients or in a critical care setting where these patients typically receive treatment. Given the lack of comprehensive information on these important patient populations, we propose to conduct a systematic literature review (SLR) evaluating recent studies on the burden of CR GN infections among high-risk patients.

## Methods

We conducted a SLR and meta-analysis of published literature to evaluate the burden of CR GN infections in patients classed as ‘high-risk’, including but not limited to those receiving treatment for cancer or receiving an organ transplant, those with renal impairment, and severely ill patients such as those with sepsis or septic shock, and in critical care settings e.g., intensive care units (ICU), skilled nursing facilities, long-term care facilities.

### Data sources and study eligibility

A comprehensive search to evaluate contemporary literature was conducted in the MEDLINE®, Cochrane Central, and Embase® databases from January 2010 through March 2019 for citations that included terms related to pathogens (e.g., Klebsiella, Pseudomonas, Escherichia), mode of infection (e.g., nosocomial, hospital-acquired), carbapenem resistance (e.g., [carbapenem, imipenem, meropenem] and resistant), treatment setting (e.g., hospital, ICU, intensive, critical, skilled nursing, long-term acute care), and patient characteristics (e.g., transplant, chronic renal insufficiency, malignancy, ventilator-dependent, immunocompromised, sepsis, bacteremia, septicemia, septic shock) (Additional Table 1; Additional Table 2].

All citations identified by literature searches were independently screened by at least two researchers using abstrackr [21]. Training sessions were implemented where all researchers screened the same articles until all team members reached consensus on the application of the eligibility criteria. Full-text publications for citations that met the inclusion criteria were retrieved and screened in duplicate. Any disagreements that arose during citation or full-text screening were resolved through discussion.

### Study inclusion criteria

We included various study types and designs (prospective and retrospective, with or without a comparator group) conducted in hospitalized adults (≥18 years) infected with CR GN pathogens. If studies included a comparator group, this could comprise patients with a ‘CS infection’ or with ‘no infection’ (i.e., patients were colonized). In addition, some studies included a comparator group where patients were confirmed not to have a CR infection, but with no explicit statement as to whether they were colonized or had a CS infection; in these instances the comparator was classified as ‘no CR infection’. Studies including patients with healthcare-associated infections (including hospital-acquired/nosocomial infections) in hospitals, nursing facilities, critical care units, or ICUs, in the US, Canada, and Western Europe (economically well-developed European nations including Germany, UK, Italy, France, Spain, Portugal, Belgium, Netherlands, and Luxembourg) were included. Outcomes of interest included all-cause mortality, infection-related mortality, length of stay in hospital or ICU, readmission, mechanical ventilation, adverse events such as nephrotoxicity, and economic outcomes such as direct costs (associated with treatment and length of stay) and total costs.

### Study exclusion criteria

We excluded abstract-only publications, cross-sectional studies, case reports, case series, narrative reviews, and any studies published before 2010. The rationale for excluding studies published prior to 2010 was to keep studies as similar as possible with regards to the definitions of carbapenem resistance and the epidemiology of the causative pathogens. We excluded studies that did not provide results for adults, healthcare-associated infections, countries of interest, or CR infections. Studies including patients with > 20% Gram-positive co-infections, reporting on specific antimicrobial therapies, or not reporting results for at least one of the outcomes of interest were excluded. We also excluded studies that included only colonized patients and did not include separate results for patients with CR infections.

### Data extraction

A customized data extraction form was created in Microsoft Excel™ to gather relevant data elements from included studies. The data extraction form was tested on several studies and revised before full data extraction. Extracted data included variables addressing study design features, enrolled and analyzed sample sizes, study population characteristics, infection characteristics, description of exposure and comparator groups, relevant outcomes, results (percentages, univariate, multivariate), and factors to inform the risk of bias assessment. Any data missing or unavailable in the publications were deemed ‘not reported’. Data from each study were extracted independently by one of three investigators and confirmed by at least one other. Any data discrepancies were identified and resolved through discussion.

### Analysis

When necessary, unadjusted odds ratios (OR) were calculated. For each outcome, meta-analyses were conducted when basic criteria were met. For effect size measures (i.e., OR, hazard ratio [HR]), if at least three studies (or independent cohorts) provided sufficient data, standard random effects model meta-analyses were performed capturing the chi-squared *P* value (values < 0.10 were deemed to be significant) and the I^2^ statistic. For the purpose of meta-analysis, all effect size measures (OR, HR, relative risk [RR]) were treated as being equivalent. Subgroup meta-analyses were also performed by length of follow-up (in hospital and ≤ 30 day follow-up, > 30 day follow-up), CR pathogen (*Klebsiella pneumoniae*, *P. aeruginosa*), comparator arm (e.g., CS infection, no infection), and high-risk ‘types’ (e.g., transplant, chronic renal insufficiency, malignancy). If a study reported results for more than one comparator group then priority in analyses was given to CS, followed by no CR, and then no infection. If a study reported results for both ≤30 day and > 30 day mortality, then ≤30 day was used in the analyses. All reported comparators and lengths of follow-up were included in the respective subgroup analyses. Studies that were heterogeneous in terms of outcome definitions were not combined in a meta-analysis, but were descriptively summarized as part of this systematic review. All analyses were performed in Stata version 13 (StataCorp, College Station, Texas).

### Risk of bias/quality assessment

Two reviewers independently assessed the risk of bias for each included study. Reviewers used the Agency of Healthcare Research and Quality (AHRQ) Risk of Bias assessment and assessed the following methodological quality items: selection bias, performance bias, detection bias, information bias, and appropriate outcome measurements. Each quality item was rated as Low, High, or Unclear.

## Results

**Fig. 1:**
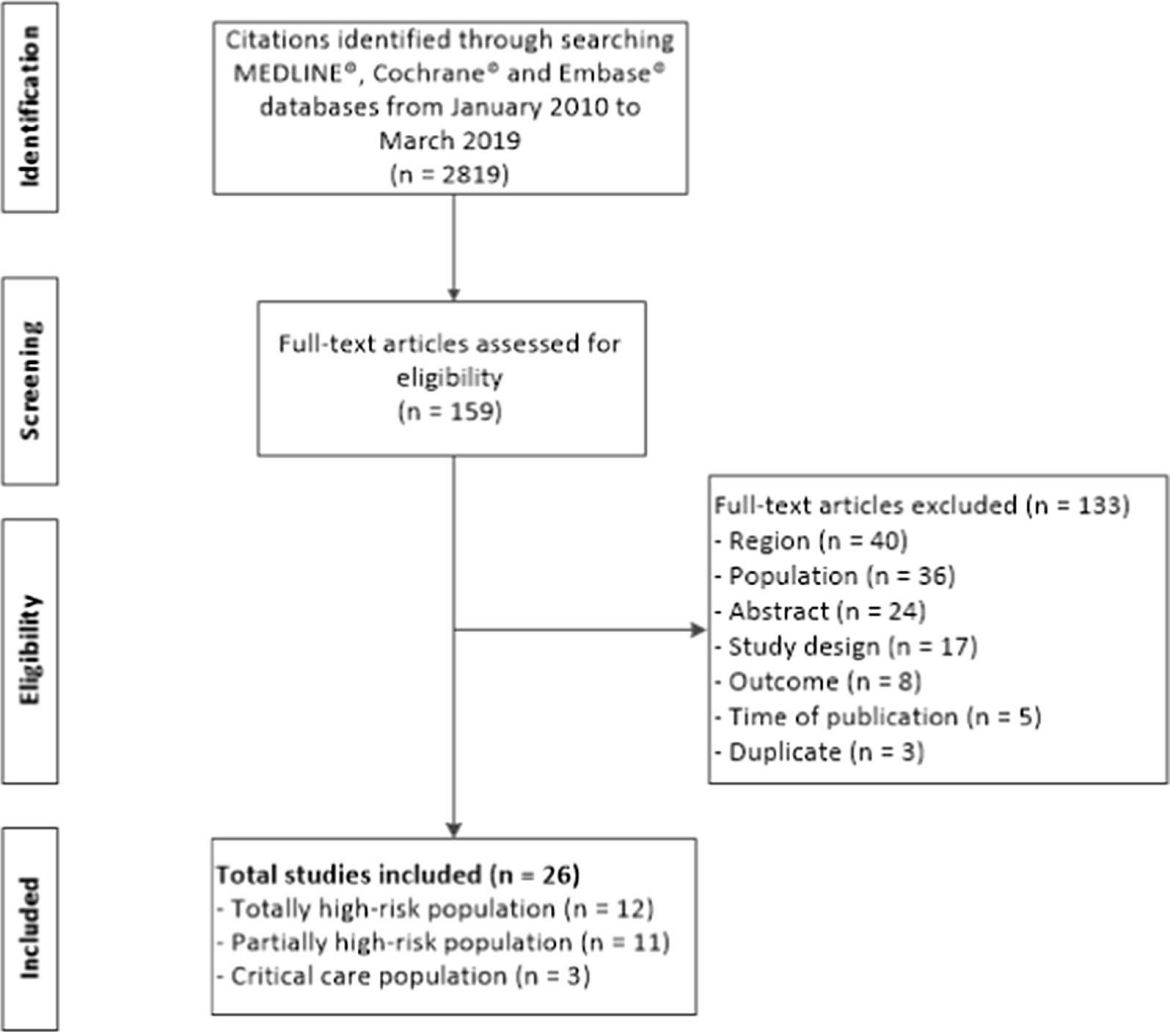
Study flow diagram. Flow diagram detailing the number of studies included and excluded at each step of the systematic review search and selection process

The literature search (Additional Table 1; Additional Table 2) identified 2819 citations, and 159 abstracts were eligible for full-text screening (Fig. 1). Full-text publications were retrieved and 133 publications were excluded during full-text screening (Additional Table 3). A total of 26 publications met the inclusion criteria (Table 1); of these, 12 studies included only high-risk patients: eight studies solely included patients who were solid organ transplant recipients, three only included patients with hematologic malignancies, and one study only included patients who had undergone open-heart surgery. The remaining 14 studies did not solely focus on high-risk patients; 11 included a subset of high-risk patients within the total population, and three included a general population of critical care patients. Twenty-two studies included patients infected with *K. pneumoniae*, two included patients infected with *P. aeruginosa*, and two focused on mixed Enterobacteriaceae infections. Sixteen studies compared at least one outcome of interest in patients with CR infection, with patients that had a CS infection, no CR infection, or no infection, while the remainder only reported results for patients with CR infection (Additional Table 4). Eight studies were prospective observational studies and the remainder were retrospective observational studies. Fourteen studies were conducted in the US, 11 in Italy, and one in Spain. Four studies received government funding, five studies received funding from various entities, eight studies reported not having received any funding, and nine studies did not report a funding source. Most studies were conducted in tertiary teaching/academic hospitals; two studies did not report this information. The sample size across the included studies ranged from 18 patients to 632 patients. The median age of included patients ranged from 51 years to 74 years, and the proportion of male study subjects ranged from 35.7 to 81.0%.

**Table 1:** Baseline characteristics of included studies

Author, year (PMID)	Country (funding source)	Setting of care	N (CR n)	Patient type (high-risk type)	Pathogen species	Age (SD/IQR)	Male (%)	Co-morbidity score	Transplant (%); diabetes (%); immunosuppression (%); renal disease (%)	Mortality (% CR; % comparator)	Other outcomes reported
Alicino 2015 (26464061) [22]	Italy (none)	Tertiary teaching hospital	489 (327)	Critical care^a^ population (NR)	*K. pneumoniae*	Median 68 (IQR 57–76)	66.2	NR	NR; NR; NR; NR	30-day (36; 23.5)	NA
Brizendine 2015 (25385105) [13]	US (NR)	Tertiary teaching hospital	108 (22)	Totally high-risk (transplant)	*K. pneumoniae*, ESBL-*K. pneumoniae*	Mean 53 (±12.1)	39	NR	100; NR; 100; NR	In-hospital (18; 2)	NA
Capone 2013 (23137235) [23]	Italy (gov.)	Tertiary teaching hospitals (one long-term facility)	97 (97)	Partially high-risk (44.3% SOT, corticosteroid therapy, immunodeficiency)	*K. pneumoniae*	Median 69 (IQR 50–77)	61.9	Charlson comorbidity index: median 5 (IQR 3–8); APACHE II: median 15 (IQR 12–20)	NR; 35; 44.3; 28.9 (CKD)	In-hospital (27.5; NA)	NA
Clancy 2013 (24011185) [24]	US (acad., gov.)	Tertiary teaching hospital	17 (17)	Totally high-risk (transplant)	*K. pneumoniae*	Median 51 (IQR 25–70)	59	APACHE II: median 18 (IQR 4–26)	100; NR; 100; 58.8	30-day (18; NA); 90-day (47; NA)	AE
Cristina 2018 (28668656) [25]	Italy (none)	Tertiary hospitals	213 (213)	Partially high-risk (14% solid malignancy, 8.5% HM, 3.3% SOT)	*K. pneumoniae*	Median 72 (IQR 61–78)	65.3	Charlson comorbidity index: median 2 (IQR 1–3)	3.29; 8.92; NR; 9.86 (chronic renal failure)	15-day (26.3; NA)	NA
Giannella 2018 (28842283) [26]	Italy (none)	Tertiary teaching hospital	595 (595)	Partially high-risk (16% chemotherapy)	*K. pneumoniae*	Median 66 (IQR 54–76)	62	Charlson comorbidity index: 3; APACHE III: 20	NR; NR; NR; 17.6 (chronic renal failure), 12.3 (hemodialysis)	14-day (21.3; NA); in-hospital (29.6; NA)	NA
Gomez-Simmonds 2015 (25878348) [27]	US (none)	Tertiary teaching hospital	223 (29)	Partially high-risk (11% SOT)	*K. pneumoniae*	Median 62; > 65: 44%	57	Charlson comorbidity index ≥4: 30%; Pitt bacteremia ≥4: 31%	15; NR; NR; 5 (ESRD requiring chronic dialysis)	30-day (41.4; 20.6)	NA
Hauck 2016 (26850824) [28]	US (acad., gov.)	Tertiary hospital	483 (260)	Partially high-risk (18% any malignancy)	*K. pneumoniae*	BSI: median 63 (IQR 54–78); pneumonia: median 68 (IQR 58–81); UTI: median 69 (IQR 57–82); control: median 71 (IQR 63–81)	42	Charlson comorbidity index: median 3 (IQR 2–5)	NR; 49.9; NR; 25.9 (renal failure [creatinine > 2 mg/dL upon admission])	In-hospital (BSI: 38; UTI: 7; pneumonia: 34; control: 9)	LOS
Hoxha 2016 (26319590) [29]	Italy (none)	NR	98 (49)	Critical care^a^ (2% transplant)	*K. pneumoniae*	CRKP: median 72; CSKP: median 74	65	Charlson comorbidity index ≥3: CRKP: 61%, CSKP: 59%	1; NR; 36; 16 (dialysis)	30-day (61; 20)	NA
Judd 2016 (27320901) [30]	US (NR)	Tertiary hospital	382 (32)	Critical care^a^ population (NR)	*P. aeruginosa*	Mean 67.2 (±14.2)	62.6	NR	NR; NR; NR; NR	In-hospital (28.1; 8.9)	Cost, LOS
Kalpoe 2012 (22467548) [31]	US (NR)	Tertiary hospital	175 (14)	Totally high-risk (liver transplant)	*K. pneumoniae*	Median 55 (IQR 23–78)	81	MELD: median 21 (IQR 6–45)	100; 31; 2 (HIV); 6 (CKD)	1-year (71; 13.7)	NA
Mazza 2017 (28457370) [32]	Italy (NR)	NR	310 (8)	Totally high-risk (liver transplant)	*K. pneumoniae*	Median 54 (IQR 18–68)	NR	NR	100; NR; 100; 85.5 (CRRT)	In-hospital (62.5; 30.4)	LOS, AE, mechanical ventilation
Messina 2016 (26686227) [33]	US (gov., industry)	Tertiary hospital	287 (109)	Partially high-risk (12% any malignancy)	*K. pneumoniae*	Median 70 (IQR 58–81)	42	Charlson comorbidity index: median 3 (IQR 2–5); Pitt bacteremia score ≥ 4: 24%	NR; 53; 12 (malignancy); 22	NA	Readmission
Micozzi 2017 (28283020) [34]	Italy (none)	Tertiary teaching hospital	22 (10)	Totally high-risk (HM)	*K. pneumoniae*	Median 51.5 (IQR 28–68)	35.7	NR	NR; NR; 100; NR	Mortality (71.4; NA)	NA
Nguyen 2010 (20356699) [35]	US (NR)	Tertiary teaching hospital	48 (48)	Partially high-risk (42% SOT, 33% immunosuppression, 8% HIV)	*K. pneumoniae*	Median 60 (IQR 37–86)	67	mAPACHE II: median 19 (IQR 12–35)	42 (SOT); 35; 8 (HIV); 44 (CRRT/ hemodialysis)	30-day (42; NA)	NA
Pena 2012 (22155832) [36]	Spain (gov.)	Tertiary hospital	632 (145)	Partially high-risk (25% immunosuppression, 85% solid malignancy, 15% HM, 1% HIV)	*P. aeruginosa*	Median 68 (IQR 55.5–77.5); SAPS II: mean 42.6 (±17.9)	69	Charlson comorbidity index: median 2 (IQR 1–4); SAPS II: mean 42.6 (±17.9); Pitt score ≥ 2: 43%	NR; 26; 25; 16	30-day (35; 27)	NA
Pereira 2015 (26136397) [37]	US (none)	Tertiary teaching hospital	304 (20)	Totally high-risk (liver transplant)	*K. pneumoniae*	Median 58 (IQR 51–62)	67	NR	100; 34; 100; NR	1-year (45; 18)	NA
Pouch 2015 (26341757) [38]	US (gov.)	Tertiary teaching hospital	100 (20)	Totally high-risk (kidney transplant)	*K. pneumoniae*	CRKP: median 57 (IQR 51–67); CSKP: median 54 (IQR 40–63)	42	NR	100; 27; NR; 88 (RRT), 8 (polycystic kidney disease), 100 (kidney transplant)	Mortality (30; 10)	AE
Qureshi 2014 (24637691) [39]	US (gov.)	Tertiary hospital	133 (133)	Partially high-risk (33% transplant, 9% solid malignancy, 42% HIV)	*K. pneumoniae*	ASB: median 62 (IQR 20–91); UTI: median 51 (IQR 24–67)	37.1	Charlson comorbidity index: ASB: median 4 (IQR 0–13); UTI: median 2 (IQR 0–7)	33.3; 44.8; 41.9; 16.2 (moderate to severe)	30-day (6; NA)	LOS, readmission
Salsano 2016 (27371609) [40]	Italy (NR)	Tertiary teaching hospital	553 (32)	Totally high-risk (open-heart surgery)	*K. pneumoniae*	CRKP: median 74 (IQR 67–77); no CRKP: median 71 (IQR 63–77)	68.5	Charlson comorbidity index: CRKP: median 3 (IQR 1–4); no CRKP: median 1 (IQR 1–2)	NA; 23.5; 1.4 (history of immunosuppression); 16.3 (CKD)	30-day (18.8; NA); in-hospital (25; 6)	NA
Satlin 2013 (22916826) [41]	US (found., gov.)	Tertiary teaching hospital	18 (18)	Totally high-risk (HM)	Mixed Enterobacteriaceae	Median 56 (IQR 24–77)	39	NR	33 (stem cell); NR; 100; NR	14-day (53; NA); in-hospital (56; NA)	Mechanical ventilation
Simkins 2014 (25092500) [42]	US (NR)	Tertiary teaching hospital	52 (13)	Totally high-risk (kidney transplant)	*K. pneumoniae*	CRKP: mean 53 (±18); CSKP: mean 55 (±16)	CRKP: 54; CSKP: 36	NR	100 (kidney transplant); 62 CRKP, 67 CSKP; 100; 100	6-month (38.5; 0); 6.5-month (46; 0)	AE
Sotgiu 2018 (29621600) [43]	Italy (none)	Tertiary teaching hospital	46 (46)	Partially high-risk (13% cancer)	*K. pneumoniae*	Mean 69.3 (±13.0)	67.4	NR	NR; 8.6; NR; 2.9 (chronic renal failure)	Mortality (52.3; NA)	NA
Tamma 2016 (28013264) [44]	US (acad., found., gov.)	Tertiary teaching hospital	83 (83)	Partially high-risk (3.9% HM, 12% SOT, 22.9% chemotherapy, 2.4% HIV)	Mixed Enterobacteriaceae	CP-CRE: median 58 (IQR 48–68); non-CP-CRE: median 58 (IQR 43–62)	CP-CRE: 59; non-CP-CRE: 63	Pitt bacteremia ≥4: CP-CRE: 54%; non-CP-CRE: 39%	11 CP-CRE, 13 non-CP-CRE (SOT); 5 CP-CRE, 2 non-CP-CRE (HSCT); 32 CR-CRE, 13 non-CR-CRE; NR; 8 CP-CRE, 4 non-P-CRE (ESRD)	14-day (15.7; NA); 30-day (20.5; NA)	NA
Trecarichi 2016 (27428072) [45]	Italy (NR)	Tertiary teaching hospitals	278 (161)	Totally high-risk (HM)	*K. pneumoniae*	Age > 54: 56.1%	54.3	NR	22.3 (HSCT); 12.6; 100; 4	21-day (52.2; 14.5)	NA
Varotti 2017 (28796391) [46]	Italy (NR)	Tertiary teaching hospital	82 (26)	Totally high-risk (kidney transplant)	*K. pneumoniae*	CRKP: mean 59 (±13); CRKP-ve: mean 53 (±14)	CRKP: 81; CRKP-ve: 84	Clavien Dindo: CRKP: mean 2.4 (±1.5); CRKP-ve: mean 1.5 (±1.1)	100 (kidney transplant); NR; 100; 100 (kidney transplant)	Mortality (8; NA)	AE, LOS, readmission

*Acad.* Academic, *AE* Adverse event, *APACHE* Acute Physiology and Chronic Health Evaluation, *ASB* Asymptomatic bacteriuria, *BSI* Bloodstream infection, *CKD* Chronic kidney disease, *CP-CRE* Carbapenemase-producing carbapenem-resistant Enterobacteriaceae, *CR* Carbapenem-resistant, *CRKP* Carbapenem-resistant *Klebsiella pneumoniae*, *CRRT* Continuous renal replacement therapy, *ESBL* Extended-spectrum beta-lactamase, *ESRD* End stage renal disease, *Found.* Foundation, *Gov.* Government, *HIV* Human immunodeficiency virus, *HM* Hematologic malignancies, *HSCT* Hematopoietic stem cell transplantation, *IQR* Interquartile range, *K. pneumoniae Klebsiella pneumonia*, *LOS* Length of stay, *MELD* Model for End-Stage Liver Disease, *N* Number, *NA* Not applicable, *NR* Not reported, *P. aeruginosa Pseudomonas aeruginosa*, *RRT* Renal replacement therapy, *SAPS* Simplified Acute Physiology, *SD* Standard deviation, *SOT* Solid organ transplant, *US* United States, *UTI* Urinary tract infection

^a^Majority were critical care population

### Mortality

Twenty-five studies reported mortality in patients with CR infections [13, 22–32, 34–46]. Of these, 12 compared mortality in patients with CR infections with mortality in patients without a CR infection (e.g., CS infection, no CR infection, no infection), which permitted the calculation of unadjusted ORs [13, 22, 27–30, 32, 36–38, 42, 45], 11 provided results for patients with CR infection only [23–26, 34, 35, 39, 41, 43, 44, 46], and two [31, 40] reported 30-day mortality for patients with CR infection only and either 6-month or 1-year mortality for both patients with CR infection and patients with no CR infection. Mortality was reported across pathogen types and type of comparator arm. Twelve studies reported data for high-risk patients exclusively (solid organ transplant, hematologic malignancy, and open-heart surgery patients); of these, eight studies compared mortality in CR infected patients with controls [13, 31, 32, 37, 38, 40, 42, 45], and six studies provided results for the CR infection patients only [24, 31, 34, 40, 41, 46] (two studies reported both comparative and non-comparative data, depending on the outcome).

#### Meta-analysis of mortality data from studies of totally high-risk patient populations

**Fig. 2:**
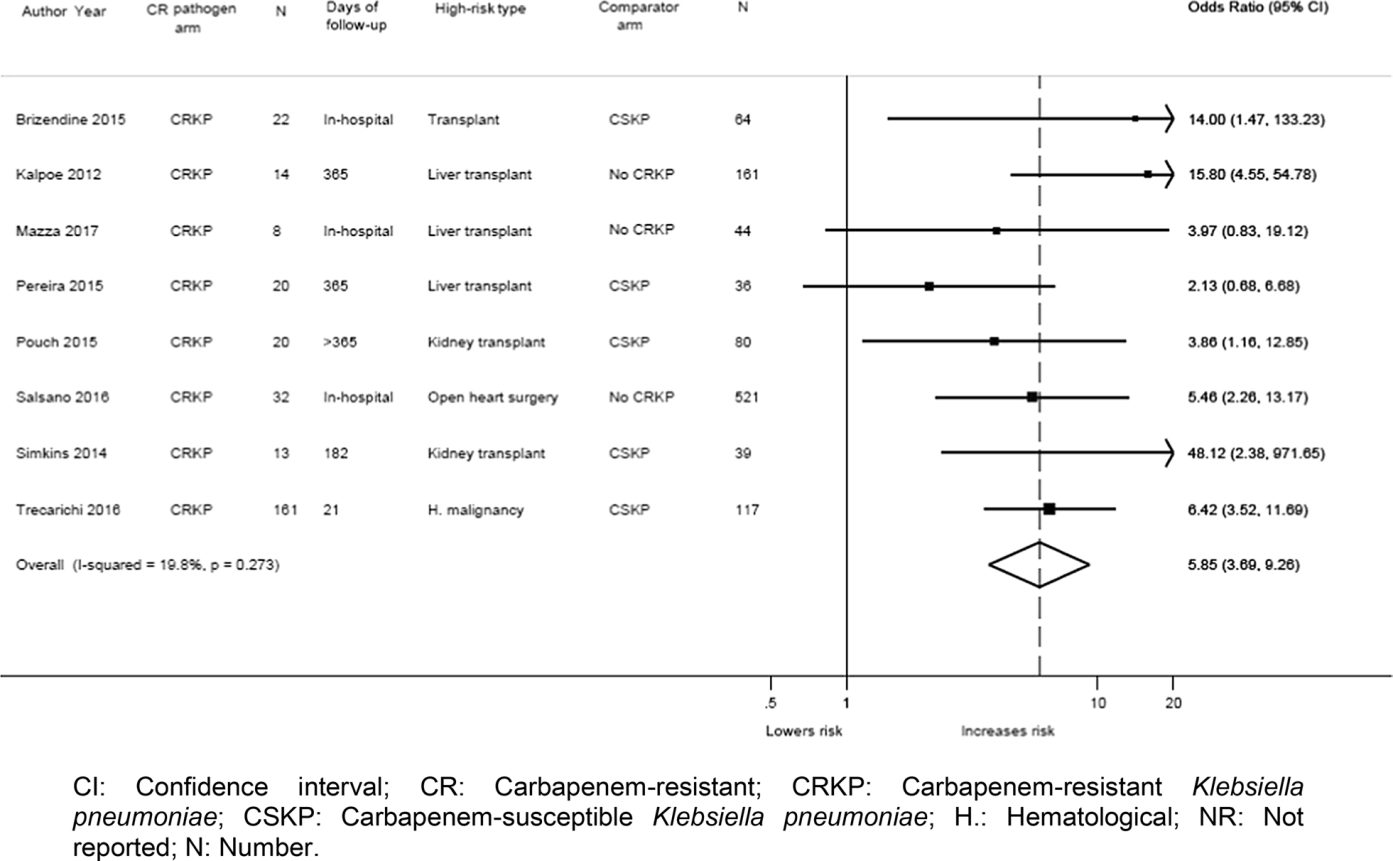
Forest plot of unadjusted mortality data from studies of totally high-risk patient populations. Forest plot that displays a significant increase in mortality risk for high-risk patients with CRKP infections compared to controls (either patients with CSKP or patients without an infection) among the eight studies that reported unadjusted mortality data. CI: Confidence interval; CR: Carbapenem-resistant; CRKP: Carbapenem-resistant *Klebsiella pneumoniae*; CSKP: Carbapenem-susceptible *Klebsiella pneumoniae*; H.: Hematological; NR: Not reported; N: Number

The eight studies that focused on high-risk patients only and compared CR infections with controls all provided unadjusted data, and five of these studies also reported adjusted data. Meta-analysis was conducted using the eight studies which reported unadjusted data [13, 31, 32, 37, 38, 40, 42, 45] and found a significant increase in mortality risk for high-risk patients with CR *K. pneumoniae* (CRKP) infections (*n* = 290) (summary OR: 5.85; 95% CI: 3.69, 9.26; I^2^ = 19.8%) compared to controls (either patients with CS *K. pneumoniae* [CSKP] or patients without an infection [*n* = 1062]) (Fig. 2). In sensitivity analysis, excluding two studies [31, 42] with an unclear risk of bias (Additional Table 6), the analysis still found a significantly increased risk of mortality among patients with CR infections compared to controls (summary OR: 5.07; 95% CI: 3.38, 7.59; I^2^ = 0.0%). Subgroup analysis by CRKP vs. CSKP (5 studies [13, 37, 38, 42, 45]; summary OR: 5.24; 95% CI: 2.65, 10.37; I^2^ = 32.1%), CRKP vs. patients who did not become infected with CRKP (no CR infection) (3 studies [31, 32, 40]; summary OR: 7.02; 95% CI: 3.33, 14.80; I^2^ = 18.4%), and CRKP vs. no infection (2 studies [32, 37]; summary OR: 24.76; 95% CI: 4.14, 148.00; I^2^ = 71.4%) found that there was a significantly higher risk of mortality in patients with CR infections irrespective of the comparator (Table 2). Additional subgroup meta-analysis of unadjusted mortality data at different time points similarly found a significantly higher risk of mortality in patients with CR infections than in controls: in-hospital/≤30-day mortality (4 studies [13, 32, 40, 45]; summary OR: 6.08; 95% CI: 3.83, 9.66; I^2^ = 0.0%) and longer follow-up (greater than 6 months) (5 studies [31, 37, 38, 40, 42]; summary OR: 6.56; 95% CI: 2.81, 15.33; I^2^ = 51.3%) (Table 2).

**Table 2:** Meta-analysis results using calculated unadjusted ORs

Analysis for unadjusted mortality data	Studies (N)	Calculated OR (95% CI)
Totally high-risk patient populations	8	5.85 (3.69, 9.26); I^2^ = 19.8%
Low risk of bias	6	5.07 (3.38, 7.59); I^2^ = 0.0%
CS infection comparator	5	5.24 (2.65, 10.37); I^2^ = 32.1%
No CR infection comparator	3	7.02 (3.33, 14.80); I^2^ = 18.4%
No infection comparator	2	24.76 (4.14, 148.00); I^2^ = 71.4%
In-hospital/≤30-day mortality	4	6.08 (3.83, 9.66); I^2^ = 0.0%
Longer-term (> 30 days) mortality	5	6.56 (2.81, 15.33); I^2^ = 51.3%
Italy	3	5.86 (3.65, 9.41); I^2^ = 0.0%
USA	5	5.40 (2.58, 11.30); I^2^ = 34.1%
Partially and totally high-risk patient populations	14	4.13 (2.70, 6.31); I^2^ = 71.5%
Low risk of bias	9	3.94 (2.32, 6.68); I^2^ = 71.4%
CS infection comparator	10	3.39 (2.11, 5.45); I^2^ = 70.2%
No CR infection comparator	3	7.02 (3.33, 14.80); I^2^ = 18.4%
No infection comparator	3	13.39 (4.09, 43.87); I^2^ = 75.0%
In-hospital/≤30-day mortality	10	3.74 (2.37, 5.89); I^2^ = 74.2%
Longer-term (> 30 days) mortality	5	6.56 (2.81, 15.33); I^2^ = 51.3%
CRKP studies only	12	4.68 (3.03, 7.23); I^2^ = 61.0%
CRPA studies only	2	2.25 (0.84, 6.03); I^2^ = 77.6%
Italy	5	4.21 (2.17, 8.14); I^2^ = 73.3%
USA	9	3.83 (2.54, 5.76); I^2^ = 28.1%

*CI* Confidence interval, *CRKP* Carbapenem-resistant *Klebsiella pneumoniae*, *CRPA* Carbapenem-resistant *Pseudomonas aeruginosa*, *CS* Carbapenem-susceptible, *N* Number, *OR* Odds ratio

**Fig. 3:**
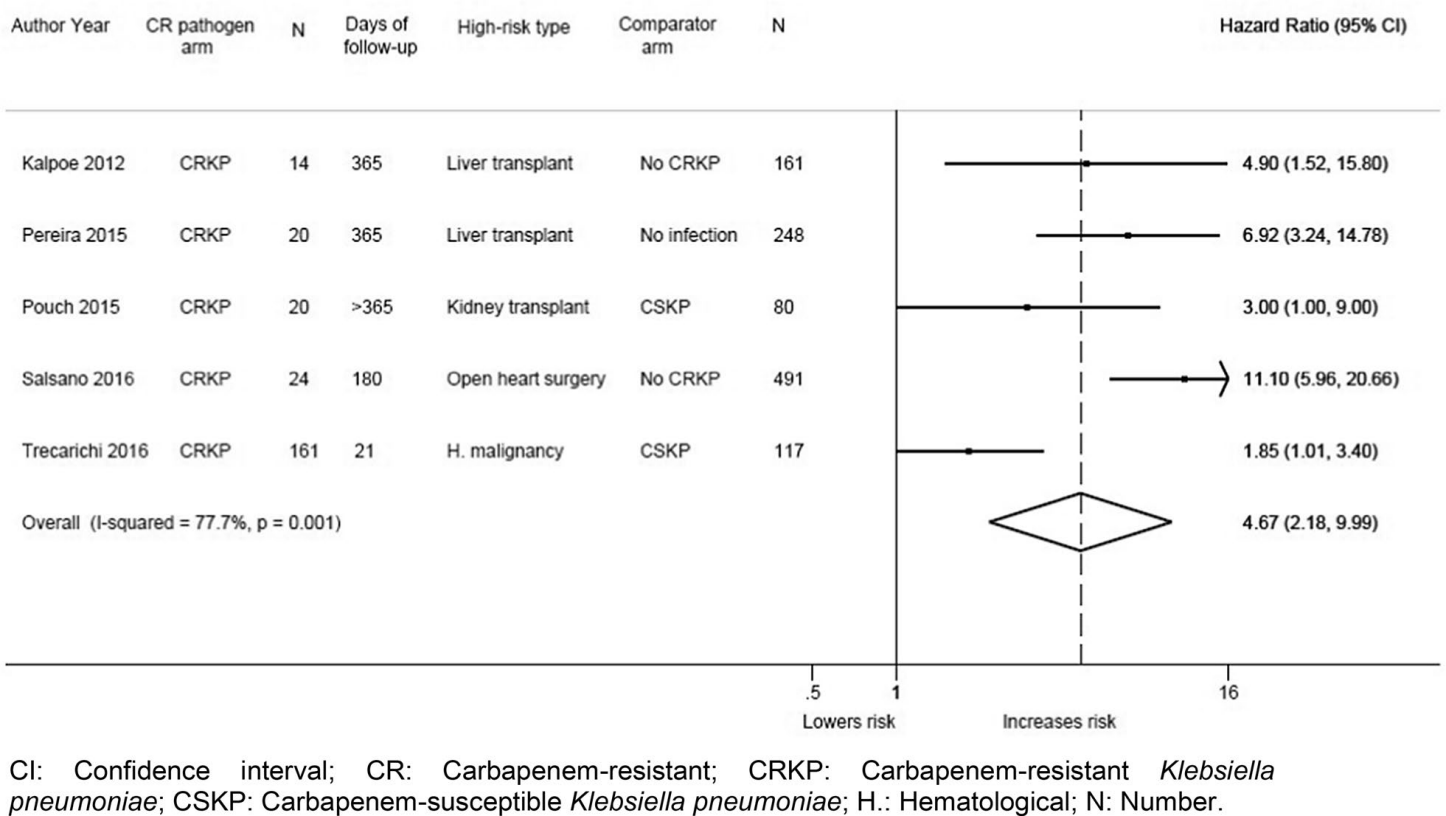
Forest plot of adjusted mortality data from studies of totally high-risk patient populations. Forest plot that displays a significant increase in mortality risk for high-risk patients with CRKP infections compared to controls (either patients with CSKP or patients without an infection) among the five studies that reported adjusted mortality data. CI: Confidence interval; CR: Carbapenem-resistant; CRKP: Carbapenem-resistant *Klebsiella pneumoniae*; CSKP: Carbapenem-susceptible *Klebsiella pneumoniae*; H.: Hematological; N: Number

Meta-analysis of the five studies that also reported multivariable data [31, 37, 38, 40, 45] (adjusted for various confounders (Additional Table 5)) compared 239 patients with CR infections to 1097 controls and found a significantly increased risk of mortality (summary HR: 4.67; 95% CI: 2.18, 9.99; I^2^ = 77.7%) (Fig. 3). In sensitivity analysis, excluding one study with an unclear risk of bias [31], the analysis also found a significantly increased risk of mortality (summary HR: 4.62; 95% CI: 1.87, 11.42; I^2^ = 83.3%). In sensitivity analysis, excluding one potential outlier study by Salsano et al. [40] (considered as such due to its large CI and exclusion of < 30 day deaths from the 180-day follow-up analysis), the analysis still found a significantly increased mortality among patients with CR infections compared to controls (summary HR: 3.57; 95% CI: 1.79, 7.14; I^2^ = 60.4%). Studies reported a significantly higher risk of mortality in patients with CR infections irrespective of the comparator. The risk of mortality was also found to be significantly increased in high-risk patients compared with controls in subgroup meta-analysis of four studies reporting adjusted data at longer follow-up periods (> 30 days) (4 studies [31, 37, 38, 40]; summary HR: 6.67; 95% CI: 3.88, 11.49; I^2^ = 0.0%) (Table 3). Only one study reported adjusted in-hospital/≤30-day mortality data [45]; mortality was significantly increased with CRKP compared to CSKP (HR: 1.85; 95% CI: 1.01, 3.40) (Table 3).

**Table 3:** Meta-analysis results using reported multivariable adjusted data

Analysis for adjusted mortality data	Studies (N)	Adjusted HR (95% CI)
Totally high-risk patient populations	5	4.67 (2.18, 9.99); I^2^ = 77.7%
Low risk of bias	4	4.62 (1.87, 11.42); I^2^ = 83.3%
CS infection comparator	2	2.07 (1.22, 3.53); I^2^ = 0.0%
No CR infection comparator	2	8.63 (4.12, 18.08); I^2^ = 31.6%
No infection comparator	1	6.92 (3.24, 14.79)
Longer-term (> 30 days) mortality	4	6.67 (3.88, 11.49); I^2^ = 0.0%
Italy	2	4.53 (0.78, 26.20); I^2^ = 93.3%
USA	3	5.19 (2.99, 9.01); I^2^ = 0.0%
Partially and totally high-risk patient populations	8	3.38 (1.93, 5.94); I^2^ = 76.0%
Low risk of bias	6	3.87 (2.10, 7.13); I^2^ = 76.9%
CS infection comparator	4	1.93 (1.24, 2.99); I^2^ = 8.2%
No CR infection comparator	2	8.63 (4.12, 18.08); I^2^ = 31.6%
No infection comparator	2	4.08 (1.56, 10.65); I^2^ = 76.5%
In-hospital/≤30-day mortality	4	2.05 (1.39, 3.02); I^2^ = 16.9%
Longer-term (> 30 days) mortality	4	6.67 (3.88, 11.49); I^2^ = 36.6%
CRKP studies only	7	3.45 (1.83, 6.51); I^2^ = 79.3%
Studies reporting HR	6	4.17 (2.23, 7.80); I^2^ = 76.6%
Studies reporting OR	2	1.71 (0.60, 4.82); I^2^ = 60.1%
Italy	2	4.53 (0.78, 26.20); I^2^ = 93.3%
USA	6	3.02 (1.79, 5.08); I^2^ = 54.2%

*CI* Confidence interval, *CRKP* Carbapenem-resistant *Klebsiella pneumoniae*, *CS* Carbapenem-susceptible, *HR* Hazard ratio, *N* Number, *OR* Odds ratio

#### Meta-analysis of mortality data from all studies (totally and partially high-risk patient populations)

**Fig. 4:**
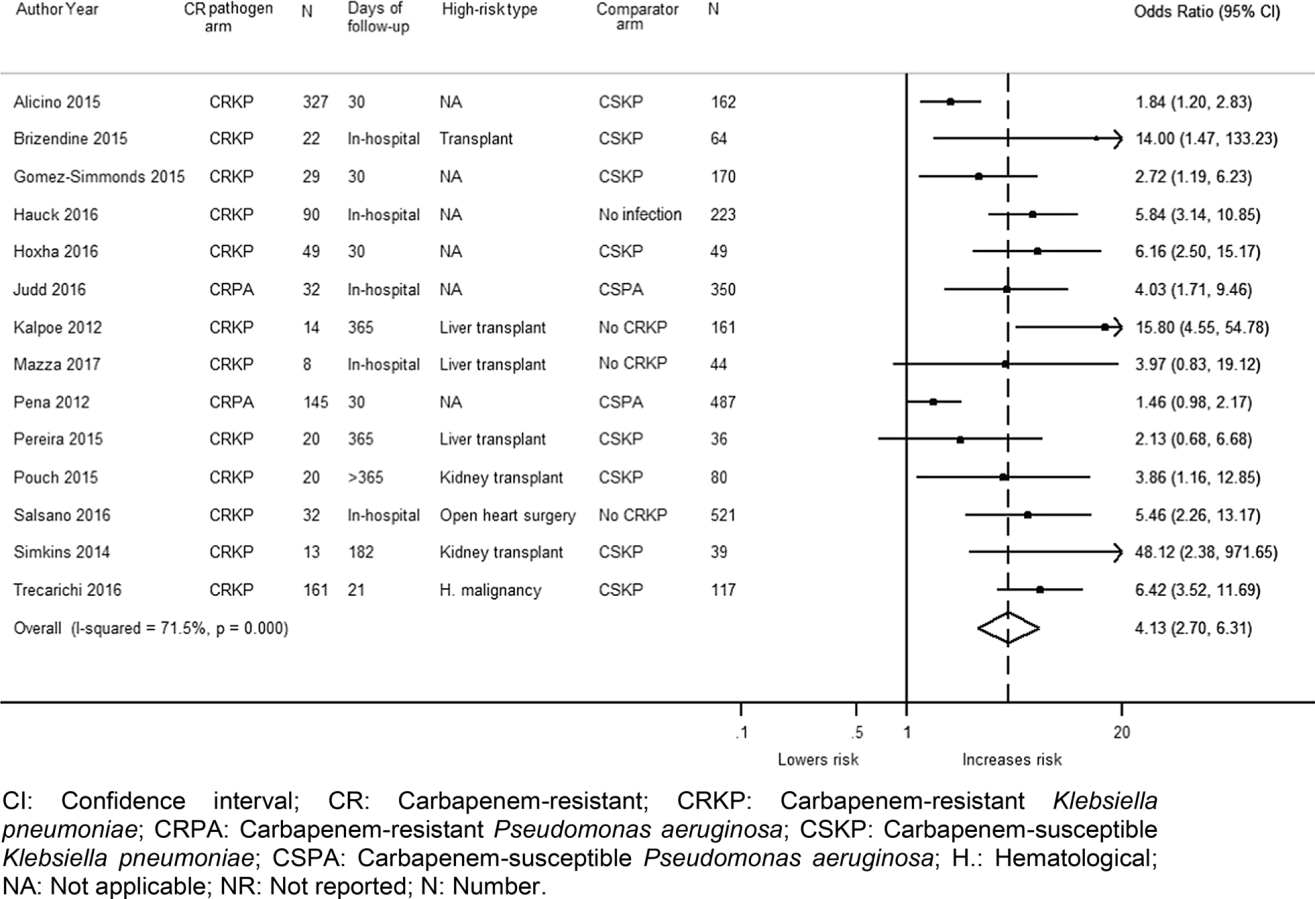
Forest plot of unadjusted mortality data from all studies (both totally and partially high-risk patients). Forest plot that displays a significant increase in mortality risk in patients with CRKP or CRPA infections compared to controls (either patients with CSKP or patients without an infection) among the 14 studies that reported unadjusted mortality data. CI: Confidence interval; CR: Carbapenem-resistant; CRKP: Carbapenem-resistant *Klebsiella pneumoniae*; CRPA: Carbapenem-resistant *Pseudomonas aeruginosa*; CSKP: Carbapenem-susceptible *Klebsiella pneumoniae*; CSPA: Carbapenem-susceptible *Pseudomonas aeruginosa*; H.: Hematological; NA: Not applicable; NR: Not reported; N: Number

Meta-analysis of 14 studies reporting unadjusted data [13, 22, 27–32, 36–38, 40, 42, 45] found a significant increase in the risk of mortality for patients with CR GN infections (*n* = 1132) compared to controls (*n* = 2949) (summary OR: 4.13; 95% CI: 2.70, 6.31; I^2^ = 71.5%) (Fig. 4). In sensitivity analysis, excluding five studies [22, 27, 29, 31, 42] with high or unclear risk of bias (Additional Table 6), the analysis still found a significantly increased risk of mortality among patients with CR infections compared to controls (summary OR: 3.94; 95% CI: 2.32, 6.68; I^2^ = 71.4%). Subgroup analyses by CR vs. CS (10 studies [13, 22, 27, 29, 30, 36–38, 42, 45]; summary OR: 3.39; 95% CI: 2.11, 5.45; I^2^ = 70.2%), CRKP vs. patients who did not develop CRKP (3 studies [31, 32, 40]; summary OR: 7.02; 95% CI: 3.33, 14.08; I^2^ = 18.4%), or CRKP vs. no infection (3 studies [28, 32, 37]; summary OR: 13.39; 95% CI: 4.09, 43.87; I^2^ = 75.0%) all found that there was a significantly higher risk of mortality in patients with CR infections irrespective of the comparator (Table 2). Additional subgroup meta-analysis of unadjusted mortality data at different time points similarly found a significantly higher risk of mortality in patients with CR infections than in controls: in-hospital/≤30-day mortality (10 studies [13, 22, 27–30, 32, 36, 40, 45]; summary OR: 3.74; 95% CI: 2.37, 5.89; I^2^ = 74.2%) and longer follow-up (greater than 30 days) (5 studies [31, 37, 38, 40, 42]; summary OR: 6.56; 95% CI: 2.81, 15.33; I^2^ = 51.3%) (Table 2). Finally, mortality is significantly increased irrespective of the pathogen type: CRKP studies only (12 studies [13, 22, 27–29, 31, 32, 37, 38, 40, 42, 45]; summary OR: 4.68; 95% CI: 3.03, 7.23; I^2^ = 61.0%) and carbapenem-resistant *P. aeruginosa* (CRPA) studies only (2 studies [30, 36]; summary OR: 2.25; 95% CI: 0.84, 6.03; I^2^ = 77.6%) (Table 2).

Meta-analyses of three studies including patients that were not categorized as high-risk, but who were treated in a critical care setting (408 patients with CR infections and 561 controls) found an increase in the risk for mortality (3 studies [22, 29, 30]; summary OR: 3.30; 95% CI: 1.52, 7.14; I^2^ = 71.4%).

**Fig. 5:**
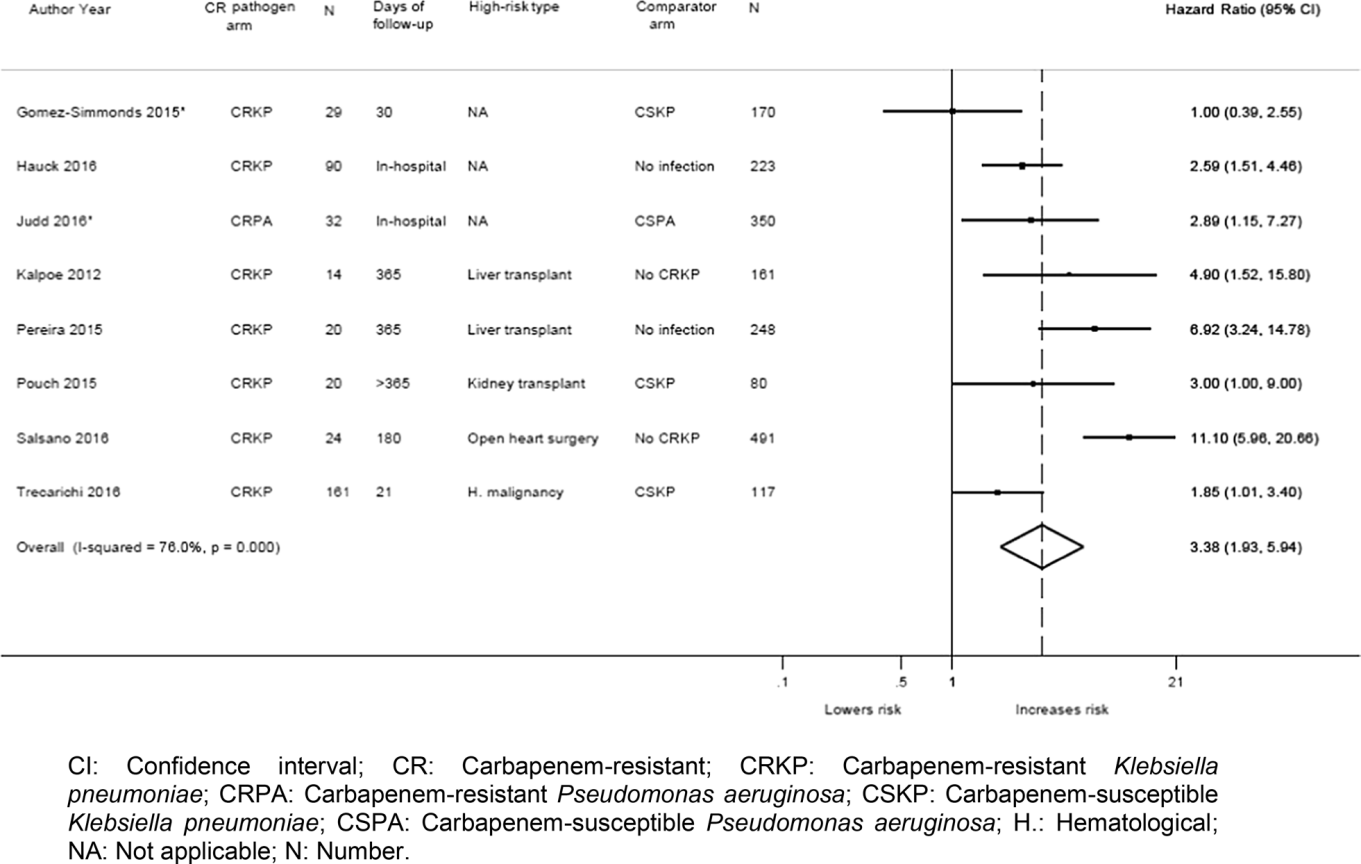
Forest plot of adjusted mortality data from all studies (both totally and partially high-risk patients). Forest plot that displays a significant increase in mortality risk in patients with CRKP or CRPA infections compared to controls (either patients with CSKP or patients without an infection) among the 8 studies that reported adjusted mortality data. CI: Confidence interval; CR: Carbapenem-resistant; CRKP: Carbapenem-resistant *Klebsiella pneumoniae*; CRPA: Carbapenem-resistant *Pseudomonas aeruginosa*; CSKP: Carbapenem-susceptible *Klebsiella pneumoniae*; CSPA: Carbapenem-susceptible *Pseudomonas aeruginosa*; H.: Hematological; NA: Not applicable; N: Number

Eight studies reported multivariate results (adjusted for various confounders (Additional Table 5)) comparing patients with CR infections (*n* = 560) to controls (*n* = 1840). Meta-analysis of the adjusted data showed a significantly increased risk of mortality for patients with CR GN infections compared to controls (8 studies [27, 28, 30, 31, 37, 38, 40, 45]; summary HR: 3.38; 95% CI: 1.93, 5.94; I^2^ = 76.0%) (Fig. 5). This significant increase was observed irrespective of the comparator type (CS infection, patients who did not develop CRKP, or no infection) (Table 3). In a sensitivity analysis, excluding two studies [27, 31] with an unclear risk of bias, the analysis also found a significantly increased risk of mortality (summary HR: 3.87; 95% CI: 2.10, 7.13; I^2^ = 76.9%).

Subgroup analysis of adjusted mortality data by duration of follow-up and by the type of pathogen similarly demonstrated significant increases in the risk for mortality. Adjusted mortality was significantly increased among patients with CR infection compared to controls for the following subgroups: in-hospital/≤30 day mortality (4 studies [27, 28, 30, 45]; summary HR: 2.05; 95% CI: 1.39, 3.02; I^2^ = 16.9%), long-term mortality (4 studies [31, 37, 38, 40]; summary HR: 6.67; 95% CI: 3.88, 11.49; I^2^ = 36.6%), and patients with CRKP infection (7 studies [27, 28, 31, 37, 38, 40, 45]; summary HR: 3.45; 95% CI: 1.83, 6.51; I^2^ = 79.3%) (Table 3). Two of the eight studies reported adjusted ORs [27, 30] and the other six reported adjusted HRs [28, 31, 37, 38, 40, 45]. Adjusted HRs were combined in subgroup analyses, showing a significantly increased risk for mortality (6 studies [28, 31, 37, 38, 40, 45]; summary HR: 4.17; 95% CI: 2.23, 7.80; I^2^ = 76.6%) (Table 3).

#### Mortality data from studies without a comparator group

Thirteen studies [23–26, 31, 34, 35, 39–41, 43, 44, 46] that did not report mortality for a comparator group reported mortality rates ranging between 8.0 and 71.4% for CR patients. Of these studies, six included only high-risk patients [24, 31, 34, 40, 41, 46]. Kalpoe 2012 [31] reported 50% 30-day mortality in liver transplant recipients with CRKP, Clancy 2013 [24] reported 47% mortality in transplant recipients with CRKP infection at 90 days, and Varotti 2017 [46] reported 8% mortality in kidney transplant patients with CRKP after at least 6 months. Micozzi 2017 [34] reported 71.4% 12-day mortality in hematological malignancy patients with CRKP and Satlin 2013 [41] reported 39% 7-day mortality, 53% 14-day mortality, and 56% in-hospital mortality in patients with hematological malignancy and bloodstream infection due to CR Enterobacteriaceae (majority with *K. pneumoniae*). Salsano 2016 [40] reported 18.8% 30-day mortality in open heart surgery patients with CRKP.

### Mechanical ventilation

Two studies reported mechanical ventilation data [32, 41]. One study [32] reported that all of the liver transplant recipients with CRKP (*n* = 8) required mechanical ventilation during the post-transplant period compared to 64% of liver transplant recipients infected with micro-organisms other than CRKP. In the second study (no comparator [41]), 11% of the 18 patients with hematologic malignancies and bloodstream infections due to CR Enterobacteriaceae required mechanical ventilation.

### Adverse events

Five studies reported adverse events; these studies all examined transplant recipients with CRKP infection [24, 32, 38, 42, 46] . Three of these studies reported graft-related outcomes in kidney transplant recipients, comparing CRKP with either CSKP patients or patients who never became CRKP positive during the follow-up period [38, 42, 46]. Only one of these studies reported a significant difference, in delayed graft function (42% vs. 17%, *p* = 0.03) for patients infected with CRKP versus patients who never became CRKP positive during the follow-up period [46]. This study also reported significantly more acute rejections in patients with CRKP infection versus patients who did not become CRKP positive (11% vs. 0%, p = 0.03). In two other studies, there was no significant difference between patients with CRKP vs. CSKP infection for either graft failure (20% vs. 16%, *p* = 0.73) [38] or graft loss (15% vs. 3%, *p* = 0.15) [42], and no difference in the rate of rejection between CRKP- and CSKP-infected patients (20% vs. 18%, *p* = 0.47) [38].

Three studies reported renal adverse events [24, 32, 38]. Mazza 2017 [32] reported a significantly higher use of renal replacement therapy among liver transplant recipients infected with CKRP compared to patients infected with micro-organisms other than CRKP (87% vs. 41%, *p* = 0.018). Clancy 2013 [24] reported that 29% of transplant patients infected with CRKP developed antibiotic-induced renal failure. The Pouch 2015 study [38] also reported that 20% of CRKP patients developed nephrotoxicity with antimicrobial therapy, but did not report results for the CSKP arm.

Finally, Varotti 2017 [46] reported significantly more medical complications in patients with CRKP infections than in patients who did not become CRKP positive (92% vs. 19%, *p* < 0.001).

### Readmission

Three studies reported readmission [33, 39, 46]. In one study reporting CRKP-infected patients [39], 38% of those with UTI and 31% of those with asymptomatic bacteriuria were readmitted within 30 days due to causes unrelated to the CKRP infection. In a second study, 20.2% of CRKP survivors were readmitted within 90 days and were found to be culture-positive for CRKP upon readmission [33]. The time period for readmission was undefined in the third study, which noted readmission in 81% of CRKP-infected kidney transplant recipients compared to 21% of kidney transplant recipients who never became CRKP positive during the follow-up [46].

### Cost

Judd and colleagues [30] compared costs for patients with meropenem-resistant or meropenem-susceptible *P. aeruginosa* (MRPA vs. MSPA) infections; for patients admitted between 2011 and 2013 the total visit cost was significantly higher (p < 0.001) for MRPA compared to MSPA (median: $37,331; range: $17,141–$77,333 vs. $15,995; range: $8542–$31,811).

### Length of stay

Six studies were identified; five reported median length of stay [28, 30, 39, 40, 46] and two reported median length of ICU stay [30, 32]. Three of the six studies included high-risk patients only [32, 40, 46] Across studies, the median length of stay was significantly longer for CRKP-infected patients compared to patients with CS infection or no infection (ranging from 10 to 41 days vs. 5.6 to 18 days, respectively), except for a subgroup of patients with UTI in one study [28] that reported no significant difference. For ICU stay specifically, one study among liver transplant recipients found a significantly longer median length of stay for CRKP-infected patients than for patients infected with a micro-organisms other than CRKP (32.5 vs. 19.5 days, *p* = 0.001), and for patients free from infections (32.5 vs. 5.6 days, p = 0.001) [32], while another study conducted among inpatients found no significant difference in ICU admissions between MRPA and MSPA infections (12 days vs. 6 days, *p* = 0.052) [30].

## Discussion

This SLR and meta-analysis of contemporary literature outlines the burden of CR GN infections among high-risk patients. To the best of our knowledge, this is the first review that focuses on high-risk patients specifically. Two prior reviews among non-high-risk hospitalized patients reported two-fold increases in mortality among patients with CR Enterobacteriaceae infections compared to CS Enterobacteriaceae [47, 48]; our analysis found an almost six-fold increase in mortality with CR GN infections in high-risk patients (5-fold increase compared to CS infection or 8-fold increase compared to no infection). The association between CR infection and increased mortality was consistent across studies solely including high-risk patients or studies including partial high-risk populations. Increased mortality was also observed irrespective of the length of follow-up (either short-term or long-term) and for different causative pathogens or underlying resistance mechanisms. In contrast, there was no association between CR GN infections and increased mortality in the three studies that did not include any specific high-risk patient groups but did include a substantial number of patients receiving treatment in an ICU.

All outcomes, with the exception of mortality, were summarized descriptively either due to insufficient numbers of studies or heterogeneity between included studies. A limited number of studies found that CR GN infections increased hospital readmissions and costs and increased the risk for mechanical ventilation. Further, this review identified studies which reported an increased risk for adverse events in high-risk patients with CR GN infections, including nephrotoxicity linked to antimicrobial treatment, and graft failure or acute rejection in solid organ transplant recipients. Considering both the increased mortality and adverse events in high-risk patients, these findings highlight the need for new treatment paradigms and novel treatments with good efficacy and tolerability profiles to achieve more favorable outcomes for patients with high unmet need.

As with any evidence synthesis approach, the limitations of the available data will transfer into limitations of the SLR. First, the studies eligible for inclusion were heterogeneous with respect to the definition of exposure, site of infection, definitions of carbapenem resistance, and types of controls. Studies reporting on *K. pneumoniae* did not address virulence, and consequently, there may have been variation in *K. pneumoniae* virulence among the studies included in the meta-analysis. Furthermore, we were limited by the definition of carbapenem resistance as provided by the study authors, so there may also be variation in the carbapenem resistance mechanism represented in the included studies (i.e. some, but not all isolates would have been carbapenemase producers). Most eligible studies did not report on the resistance mechanisms in sufficient detail. Confounding of unadjusted data in observational studies is another well-known potential source of bias. These limitations most likely contributed to the high heterogeneity in some of our meta-analyses results. Any association of an exposure with outcomes in the presence of high heterogeneity may misrepresent the true association, and therefore, the results of these meta-analyses should be interpreted with caution. We attempted to mitigate these issues through subgroup analyses, limiting analyses to similar comparators, and combining multivariate adjusted data in meta-analyses, though we acknowledge that certain limitations will persist despite these steps. Second, for all of the outcomes of interest except mortality, it was not possible to perform meta-analysis i.e., two studies might show statistically significant effects of carbapenem resistance on the outcome, but a third study was not available to permit further exploration through meta-analysis. Finally, our study results may not be generalizable to all geographical regions as studies were included from only certain regions (e.g., North America, Western Europe). Although we aimed to include studies conducted throughout Western Europe, the available studies were only from Spain and Italy, and so our results may not be generalizable to other countries in Western Europe. Despite these limitations, we believe this review addresses an important topic and has identified the relevant contemporary information to further our understanding of the unmet need associated with high-risk patients with CR GN infections.

## Conclusions

This SLR and meta-analysis indicates that CR GN infections among high-risk patients are associated with increased mortality. As carbapenem resistance becomes more widespread, and may yet be further exacerbated through misuse or overuse of existing antimicrobials, understanding the burden of CR GN infections and the patients who are most impacted will be necessary to appropriately allocate resources to target and control these resistant infections. These findings further emphasize the need for robust and data-driven antimicrobial stewardship and infection control measures in hospitals which treat high-risk patients and the continuing need for the development of effective antimicrobials with favorable efficacy and safety profiles for the treatment of CR GN infections.

## Supplementary Information

**Additional file 1: Table S1.** Search strategy conducted in OVID MEDLINE® on March 31, 2019. **Table S2.** Search strategy conducted in Embase® on March 31, 2019. **Table S3.** Reasons for exclusion of 133 articles during full-text review. **Table S4.** Comparator for each outcome by study. **Table S5.** List of confounders in studies reporting adjusted multivariable analyses. **Table S6.** Risk of bias.

## Data Availability

Data supporting the conclusions of this article is available in the Additional Material.

## Abbreviations

AHRQ: Agency of Healthcare Research and Quality
CI: Confidence interval
CR: Carbapenem-resistant
CRKP: Carbapenem-resistant *Klebsiella pneumoniae*
CRPA: Carbapenem-resistant *Pseudomonas aeruginosa*
CS: Carbapenem-susceptible
CSKP: Carbapenem-susceptible *Klebsiella pneumoniae*
GN: Gram-negative
HR: Hazard ratio
ICU: Intensive care unit
*K. pneumoniae*: *Klebsiella pneumoniae*
MRPA: Multidrug-resistant *Pseudomonas aeruginosa*
MSPA: Multidrug-susceptible *Pseudomonas aeruginosa*
OR: Odds ratio
*P. aeruginosa*: *Pseudomonas aeruginosa*
RR: Risk ratio
SLR: Systematic literature review
UK: United Kingdom
US: United States
UTI: Urinary tract infection
