# Supplementary material for: Burden of carbapenem non-susceptible infections in high-risk patients: systematic literature review and meta-analysis
Source: Antimicrob Resist Infect Control. 2020 Dec 7;9:193. doi: 10.1186/s13756-020-00858-8 (PMC7720068; doi:10.1186/s13756-020-00858-8)
Supplement: Supplementary file 1 — Additional file 1: Table S1. Search strategy conducted in OVID MEDLINE® on March 31, 2019. Table S2. Search strategy conducted in Embase® on March 31, 2019. Table S3. Reasons for exclusion of 133 articles during full-text review. Table S4. Comparator for each outcome by study. Table S5. List of confounders in studies reporting adjusted multivariable analyses. Table S6. Risk of bias. [file 13756_2020_858_MOESM1_ESM.docx]

**Additional Table 1.** Search strategy conducted in OVID MEDLINE on March 31, 2019

| **#** | **Searches** | **Results** |
| --- | --- | --- |
| 1 | exp enterobacteriaceae/ or exp Klebsiella/ or exp Pseudomonas/ or exp escherichia/ or Klebsiella pneumonia carbapenemase/ or New Delhi Metallo-beta-lactamase/ or oxacillinase.af. or carbapenemase-producing.af. or (KPC or NDM or OXA).af. | 444,778 |
| 2 | (Klebsiella or Pseudomonas or escherichia or ESBL or enterobacter$).tw. | 335,726 |
| 3 | or/1-2 | 539,225 |
| 4 | exp carbapenems/ or carbapenem.af. | 14,141 |
| 5 | (Doribax or doripenem or ertapenem or imipenem or Invanz or meropenem or Merrem or primaxin or Tienam).tw. | 13,188 |
| 6 | or/4-5 | 20,079 |
| 7 | (resistant or resistance or nonsusceptib$ or non-susceptibl$ or MDR or XDR or PDR or (multi-drug adj1 resistan$) or (multidrug adj1 resistan$)).af. | 970,213 |
| 8 | 6 and 7 | 13,135 |
| 9 | 3 and 8 | 9,215 |
| 10 | exp Care Unit, Intensive/ | 79,338 |
| 11 | exp Care, Critical/ | 55,623 |
| 12 | ((intensive and care) or (critical and care)).af. | 434,184 |
| 13 | (ICU or MICU or SICU).tw. | 48,269 |
| 14 | exp skilled nursing facilities/ | 4,179 |
| 15 | (skilled and nursing and facilit$).af. | 5,876 |
| 16 | exp long-term care/ | 25,970 |
| 17 | ((long-term and care) or (long and term and care) or (long and term and acute and care)).af. | 129,849 |
| 18 | (hospital or hospitals or hospitalized or hospitalised or in-hospital).tw. | 1,030,722 |
| 19 | exp veterans hospital/ | 6,755 |
| 20 | (nosocomial or healthcare-associated or (healthcare adj1 associated)).af. | 30,825 |
| 21 | or/10-20 | 1,488,357 |
| 22 | 9 and 21 | 4,715 |
| 23 | transplantation.sh. or transplantation.af. or transplant$.af. | 804,671 |
| 24 | exp neoplasms/ or neoplasm$.af. or malignancy.af. or cancer$.af. or carcinoma.af. | 3,949,744 |
| 25 | exp sepsis/ or sepsis.af. | 167,657 |
| 26 | exp chronic renal insufficiency/ or exp chronic renal failure/ or dialysis.af. or ESRD.af. | 233,730 |
| 27 | (exp blood circulation/ and exp infections/) or (bloodstream and infection$).tw. or (bacteremia or bacteraemia).tw. or (septicemia or septicaemia).tw. | 54,410 |
| 28 | ((ventilator and dependent) or ventilator-dependent or ventilation or ventilated or VAP or VABP).tw. | 134,295 |
| 29 | exp immunocompromised host/ or exp HIV/ | 120,086 |
| 30 | or/23-29 | 5,075,369 |
| 31 | 9 and 30 | 1,612 |
| 32 | 22 or 31 | 5,300 |
| 33 | remove duplicates from 32 | 5,223 |
| 34 | animals/ not humans/ | 4,524,329 |
| 35 | 33 not 34 | 5,164 |
| 36 | limit 35 to yr="2010-current" | 3,469 |
| 37 | limit 36 to english language | 3,173 |
| 38 | (case reports or letter or guideline or editorial or comment or in vitro or review$).pt. | 5,481,265 |
| 39 | 37 not 38 | 2,702 |
| 40 | limit 39 to "all child (0 to 18 years)" [Limit not valid in CCTR; records were retained] | 538 |
| 41 | 39 not 40 | 2,164 |

**Additional Table 2.** Search strategy conducted in Embase on March 31, 2019

| **#** | **Searches** | **Results** |
| --- | --- | --- |
| 1 | ‘multidrug resistant' OR 'multidrug resistance'/exp OR 'multidrug resistance' OR 'multi-drug resistant' OR 'multi-drug resistance' OR 'multi drug resistant' OR 'multi drug resistance' OR MDR OR 'carbapenem-resistant enterobacteriaceae'/exp OR 'carbapenemresistant enterobacteriaceae' OR 'extreme drug resistance' OR 'pan resistance' OR 'pan resistant' OR 'klebsiella pneumoniae carbapenemase' OR KPC OR 'new delhi metallo-beta-lactamase' OR NDM OR OXA OR 'non susceptible' OR 'intermediate susceptibility' OR 'intermediate resistant' OR 'carbapenemase producing' | 127,804 |
| 2 | pseudomonas OR klebsiella OR enterobacteriaceae OR enterobacter OR escherichia | 599,296 |
| 3 | #1 AND #2 | 22,707 |
| 4 | ‘blood stream infections' OR 'bacteremia'/exp OR bacteremia OR 'bacterial pneumonia'/exp OR 'bacterial pneumonia' OR 'hospital-acquired bacterial pneumonia' OR 'healthcare-associated bacterial pneumonia' OR 'ventilator-associated bacterial pneumonia' | 85,640 |
| 5 | ‘transplant' OR 'neoplasm' OR 'sepsis' OR 'chronic kidney failure' OR 'immunocompromised patient' OR 'human immunodeficiency virus infection' | 1,830,837 |
| 6 | #3 AND #4 | 3,827 |
| 7 | #3 AND #5 | 2,098 |
| 8 | 'long term care' OR 'hospital' OR 'nursing home' OR 'intensive care' | 7,582,612 |
| 9 | #6 OR #7 | 3,186 |
| 10 | #8 AND #9 | 2,436 |
| 11 | #10 AND [adult]/lim | 909 |
| 12 | [editorial]/lim OR [erratum]/lim OR [letter]/lim OR [note]/lim OR [review]/lim OR 'case report'/de OR 'clinical protocol'/de OR 'letter':it OR 'editorial':it OR 'review':it OR 'practice guidelines' OR 'in vitro study' | 8,609,917 |
| 13 | #11 NOT #12 AND (2010:py OR 2011:py OR 2012:py OR 2013:py OR 2014:py OR 2015:py OR 2016:py OR 2017:py OR 2018:py OR 2019:py) | 655 |

**Additional Table 3.** Reasons for exclusion of 133 articles during full-text review

| **Author, year (PMID)** | **Title** | **Journal** | **Reason** |
| --- | --- | --- | --- |
| Abboud 2016 (27155944) | Post-surgical mediastinitis due to carbapenem-resistant Enterobacteriaceae: Clinical, epidemiological and survival characteristics | International Journal of Antimicrobial Agents | Region |
| Alexander 2017 (28584849) | Carbapenem-resistant Enterobacteriaceae infections: Results from a retrospective series and implications for the design of prospective clinical trials | Open Forum Infectious Diseases | Population |
| Aloush 2006 (16377665) | Multidrug-resistant *Pseudomonas aeruginosa*: Risk factors and clinical impact | Antimicrobial Agents and Chemotherapy | Region |
| Amin 2013  (No PMID) | Re-emerging of colistin for treatment of nosocomial pneumonia due to gram negative multi-drug resistant pathogens in critically ill patients | Egyptian Journal of Chest Diseases and Tuberculosis | Region |
| Anesi 2016 (26766415) | Risk factors and outcomes with carbapenem-resistant Enterobacteriaceae bloodstream infections among solid organ transplant recipients | American Journal of Transplantation | Study design |
| Anesi 2018  (No PMID) | Risk factors and outcomes with extended-spectrum beta-lactamase-producing Enterobacteriaceae bloodstream infections among solid organ transplant recipients | American Journal of Transplantation | Abstract |
| Balandin-Moreno 2013 (24354959) | Tigecycline therapy for infections due to carbapenemase-producing *Klebsiella pneumoniae* in critically ill patients | Scandinavian Journal of Infectious Diseases | Study design |
| Balkan 2014 (24998423) | Blood stream infections due to OXA-48-like carbapenemase-producing Enterobacteriaceae: treatment and survival | International Journal of Infectious Diseases | Region |
| Barchiesi 2016 (27716164) | Carbapenem-resistant *Klebsiella pneumoniae* influences the outcome of early infections in liver transplant recipients | BMC Infectious Diseases | Population |
| Bartoletti 2018 (29809304) | Management of immunosuppressive therapy in liver transplant recipients who develop bloodstream infection | Transplant Infectious Disease | Population |
| Bartsch 2017 (27642178) | Potential economic burden of carbapenem-resistant Enterobacteriaceae (CRE) in the United States | Clinical Microbiology & Infection | Study design |
| Ben-David 2012 (21722257) | Outcome of carbapenem resistant *Klebsiella pneumoniae* bloodstream infections | Clinical Microbiology & Infection | Region |
| Berger 2011  (No PMID) | Effect of previous exposure to fluoroquinolones on cross resistance and gram negative sepsis mortality | Critical Care Medicine | Abstract |
| Biderman 2015 (26065630) | Multidrug-resistant *Acinetobacter baumannii* infections in lung transplant patients in the cardiothoracic intensive care unit | Clinical Transplantation | Region |
| Biehle 2015 (26618357) | Outcomes and risk factors for mortality among patients treated with carbapenems for *Klebsiella* spp. bacteremia | PLoS ONE | Population |
| Bleumin 2012 (22722020) | Carbapenem-resistant *Klebsiella pneumoniae* is associated with poor outcome in hemodialysis patients | Journal of Infection | Region |
| Bogan 2014 (24837111) | Outcomes of carbapenem-resistant Enterobacteriaceae isolation: matched analysis | American Journal of Infection Control | Population |
| Brasseur 2015  (No PMID) | A high aminoglycoside regimen associated with renal replacement therapy for the treatment of multi-drug-resistant pathogens | Annals of Intensive Care | Abstract |
| Buehrle 2016 (27821456) | Carbapenem-resistant *Pseudomonas aeruginosa* bacteremia: Risk factors for mortality and microbiologic treatment failure | Antimicrobial Agents and Chemotherapy | Outcome |
| Cano 2018 (29126110) | Risks of infection and mortality among patients colonized with *Klebsiella pneumoniae* carbapenemase-producing *K. pneumoniae*: Validation of scores and proposal for management | Clinical Infectious Diseases | Outcome |
| Chae 2018 (29300717) | Notes from the field: Investigation of carbapenemase-producing carbapenem-resistant Enterobacteriaceae among patients at a community hospital - Kentucky, 2016 | Morbidity & Mortality Weekly Report | Study design |
| Chaves 2017 (29095142) | High mortality of bloodstream infection outbreak caused by carbapenem-resistant *P. aeruginosa* producing SPM-1 in a bone marrow transplant unit | Journal of Medical Microbiology | Region |
| Cheng 2018 (30103414) | Intravenous colistin monotherapy versus combination therapy against carbapenem-resistant gram-negative bacteria infections: Meta-analysis of randomized controlled trials | Journal of Clinical Medicine | Study design |
| Chia 2018 (28962969) | Emergence of multi-drug resistant organisms (MDROs) causing Fournier's gangrene | Journal of Infection | Population |
| Chopra 2015  (No PMID) | Epidemiology and outcomes of multidrug-resistant (MDR) gram negative infections (GNIs) in older adults | Journal of the American Geriatrics Society | Abstract |
| Correa 2013 (23398691) | A hospital-based matched case-control study to identify clinical outcome and risk factors associated with carbapenem-resistant *Klebsiella pneumoniae* infection | BMC Infectious Diseases | Region |
| Cprek 2016 (26552970) | Ertapenem-containing double-carbapenem therapy for treatment of infections caused by carbapenem-resistant *Klebsiella pneumoniae* | Antimicrobial Agents & Chemotherapy | Study design |
| Cristina 2016 (27980379) | Epidemiology and biomolecular characterization of carbapenem-resistant *Klebsiella pneumoniae* in an Italian hospital | Journal of Preventive Medicine & Hygiene | Population |
| Cuzon 2011 (21343451) | Outbreak of OXA-48-positive carbapenem-resistant *Klebsiella pneumoniae* isolates in France | Antimicrobial Agents & Chemotherapy | Outcome |
| Daroukh 2014 (25022890) | Characteristics and costs of carbapenemase-producing Enterobacteria carriers (2012/2013) | Medecine et Maladies Infectieuses | Population |
| Dautzenberg 2015 (25882764) | The association between colonization with carbapenemase-producing Enterobacteriaceae and overall ICU mortality: an observational cohort study | Critical Care Medicine | Region |
| de Maio Carrilho 2016 (27809803) | A prospective study of treatment of carbapenem-resistant Enterobacteriaceae infections and risk factors associated with outcome | BMC Infectious Diseases | Region |
| De Pascale 2017 (28679413) | Double carbapenem as a rescue strategy for the treatment of severe carbapenemase-producing *Klebsiella pneumoniae* infections: a two-center, matched case-control study | Critical Care | Population |
| Dubrovskaya 2013 (23959321) | Risk factors for treatment failure of polymyxin B monotherapy for carbapenem-resistant *Klebsiella pneumoniae* infections | Antimicrobial Agents & Chemotherapy | Study design |
| Esterly 2012 (22777044) | Evaluation of clinical outcomes in patients with bloodstream infections due to Gram-negative bacteria according to carbapenem MIC stratification | Antimicrobial Agents & Chemotherapy | Population |
| Falagas 2014 (24959688) | Deaths attributable to carbapenem-resistant Enterobacteriaceae infections | Emerging Infectious Diseases | Study design |
| Fanci 2017  (No PMID) | Multidrug-resistant gram negative bacteremia in hematologic patients: Incidence and infection control practices in a monocentric experience | Haematologica | Abstract |
| Ferstl 2017 (28374901) | The impact of carbapenem resistance on clinical deterioration and mortality in patients with liver disease | Liver International | Population |
| Ferstl 2016  (No PMID) | Colonization and infection with carbapenem-resistant non-fermenting gram-negative bacteria are associated with rapid deterioration and mortality in patients with decompensated liver disease | Zeitschrift fur Gastroenterologie | Abstract |
| Forcina 2018 (29501780) | Clinical impact of pretransplant multidrug-resistant Gram-negative colonization in autologous and allogeneic hematopoietic stem cell transplantation | Biology of Blood and Marrow Transplantation | Population |
| Freire 2016  (No PMID) | Treatment of carbapenem resistant Enterobacteriaceae with reduced susceptibility to polymyxin among kidney transplant recipients experience during an outbreak period | Transplantation | Abstract |
| Furtado 2011 (22031077) | Prevalence and clinical outcomes of episodes of ventilator-associated pneumonia caused by SPM-1-producing and non-producing imipenem-resistant *Pseudomonas aeruginosa* | Revista da Sociedade Brasileira de Medicina Tropical | Region |
| Galanopoulos 2016 (No PMID) | Risk factors for infection and high mortality rate among patients with KPC producing *Klebsiella pneumoniae* bacteraemias in a haematology unit | Haematologica | Abstract |
| Garnica 2009 (19287908) | Factors associated with bacteremia due to multidrug-resistant Gram-negative bacilli in hematopoietic stem cell transplant recipients | Brazilian Journal of Medical and Biological Research | Region |
| Giamarellos-Bourboulis 2006 (16707252) | Multidrug resistance to antimicrobials as a predominant factor influencing patient survival | International Journal of Antimicrobial Agents | Region |
| Gomez-Simmonds 2016 (27044555) | Combination regimens for treatment of carbapenem-resistant *Klebsiella pneumoniae* bloodstream infections | Antimicrobial Agents & Chemotherapy | Study design |
| Gottin 2017  (No PMID) | Procalcitonin level in *Klebsiella pneumoniae* MDR infections in ICU | Critical Care | Abstract |
| Gutierrez-Gutierrez 2017 (28373258) | EUropean prospective cohort study on Enterobacteriaceae showing REsistance to CArbapenems (EURECA): a protocol of a European multicentre observational study | BMJ Open | Population |
| Gurntke 2014 (25224765) | Molecular epidemiology of extended-spectrum beta-lactamase (ESBL)-positive *Klebsiella pneumoniae* from bloodstream infections and risk factors for mortality | Journal of Infection and Chemotherapy | Population |
| Hattemer 2013 (23733476) | Bacterial and clinical characteristics of health care- and community-acquired bloodstream infections due to *Pseudomonas aeruginosa* | Antimicrobial Agents & Chemotherapy | Population |
| Huang 2012 (23200553) | Molecular surveillance and clinical outcomes of carbapenem-resistant *Escherichia coli* and *Klebsiella pneumoniae* infections | Journal of Microbiology, Immunology & Infection | Region |
| Hyle 2010  (21029005) | Ertapenem-resistant Enterobacteriaceae: risk factors for acquisition and outcomes | Infection Control & Hospital Epidemiology | Population |
| Jaiswal 2018 (29755703) | Gut colonization with carbapenem-resistant Enterobacteriaceae adversely impacts the outcome in patients with hematological malignancies: Results of a prospective surveillance study | Mediterranean Journal of Hematology and Infectious Diseases | Region |
| Kang 2005  (15770098) | Risk factors for antimicrobial resistance and influence of resistance on mortality in patients with bloodstream infection caused by *Pseudomonas aeruginosa* | Microbial Drug Resistance | Region |
| King 2017  (28483952) | Multicenter study of outcomes with ceftazidime-avibactam in patients with carbapenem-resistant Enterobacteriaceae infections | Antimicrobial Agents & Chemotherapy | Study design |
| Kittleson 2018  (No PMID) | Multi-drug resistant infection after heart transplantation: How serious is this? | Journal of Heart and Lung Transplantation | Abstract |
| Kofteridis 2014 (24703709) | Risk factors for carbapenem-resistant *Klebsiella pneumoniae* infection/colonization: a case-case-control study | Journal of Infection & Chemotherapy | Region |
| Landman 2012 (22378678) | Transmission of carbapenem-resistant pathogens in New York City hospitals: progress and frustration | The Journal of Antimicrobial Chemotherapy | Outcome |
| Lanini 2015 (25835018) | Incidence of carbapenem-resistant gram negatives in Italian transplant recipients: a nationwide surveillance study | PLoS ONE | Population |
| Laupland 2005 (16206075) | Population-based epidemiological study of infections caused by carbapenem-resistant *Pseudomonas aeruginosa* in the Calgary Health Region: Importance of metallo-β-lactamase (MBL)-producing strains | Journal of Infectious Diseases | Time of publication |
| Lautenbach 2010 (19951202) | Imipenem resistance in *Pseudomonas aeruginosa*: emergence, epidemiology, and impact on clinical and economic outcomes | Infection Control & Hospital Epidemiology | Population |
| Lee 2017  (26188977) | Risk factors and clinical significance of bacteremia caused by *Pseudomonas aeruginosa* resistant only to carbapenems | Journal of Microbiology, Immunology & Infection | Region |
| Lee 2017  (No PMID) | Carbapenem-resistant Enterobacteriaceae isolated from bile in patients with biliary obstruction: Epidemiology and outcome | International Journal of Antimicrobial Agents | Abstract |
| Lee 2018  (30577266) | Acquisition of carbapenemase-producing Enterobacteriaceae in solid organ transplantation recipients | Transplantation Proceedings | Region |
| Lespada 2018 (30512080) | Bacteremia caused by *Klebsiella pneumoniae* carbapenemase (KPC)-producing *K. pneumoniae*. A retrospective study of 7 years | Revista Espanola de Quimioterapia | Region |
| Lubbert 2014 (24217959) | Colonization of liver transplant recipients with KPC-producing *Klebsiella pneumoniae* is associated with high infection rates and excess mortality: a case–control analysis | Infection | Population |
| Mandelli 2018  (No PMID) | Ceftazidime-avibactam to treat severe deep-seated infections due to KPcp in the critically ill | Intensive Care Medicine Experimental | Abstract |
| Mastoraki 2008 (18426347) | *Pseudomonas aeruginosa* susceptible only to colistin in intensive care unit patients | Surgical Infections | Region |
| McConville 2017 (29023567) | Carbapenem-resistant Enterobacteriaceae colonization (CRE) and subsequent risk of infection and 90-day mortality in critically ill patients, an observational study | PLoS ONE | Population |
| Michalopoulos 2010 (19694767) | Intravenous fosfomycin for the treatment of nosocomial infections caused by carbapenem-resistant *Klebsiella pneumoniae* in critically ill patients: a prospective evaluation | Clinical Microbiology & Infection | Region |
| Michalopoulos 2010 (No PMID) | Intravenous fosfomycin for the treatment of nosocomial infections due to carbapenem-resistant *Klebsiella pneumoniae* in critically ill patients. A prospective evaluation | Clinical Microbiology & Infection | Duplicate |
| Moorhouse 2012  (No PMID) | Influence of a beta-lactam allergy on antimicrobial coverage and in-hospital mortality in gram-negative bacteremia | Critical Care Medicine | Abstract |
| Mouloudi 2014 (25420863) | Bloodstream infections caused by carbapenemase-producing *Klebsiella pneumoniae* among intensive care unit patients after orthotopic liver transplantation: risk factors for infection and impact of resistance on outcomes | Transplantation Proceedings | Region |
| Mularoni 2018  (No PMID) | Containment of carbapenem-resistant Enterobacteriaceae (CRE) infections at a solid organ transplant centre | American Journal of Transplantation | Abstract |
| Nambiar 2017  (No PMID) | Clinical experience with novel cephalosporin/beta-lactamase inhibitor combinations in the treatment of multidrug resistant *Pseudomonas aeruginosa* and carbapenem-resistant Enterobacteriaceae in solid organ transplant recipients | American Journal of Transplantation | Abstract |
| Navarro-San Francisco 2012  (No PMID) | Clinical features and outcome of 26 bloodstream infections (BSI) caused by OXA-48 producing Enterobacteriaceae in a university hospital in Spain | Clinical Microbiology and Infection | Abstract |
| Navarro-San Francisco 2012 (23231088) | Bacteraemia due to OXA-48-carbapenemase-producing Enterobacteriaceae: a major clinical challenge | Clinical Microbiology & Infection | Study design |
| Neuner 2012 (21396529) | Treatment and outcomes in carbapenem-resistant *Klebsiella pneumoniae* bloodstream infections | Diagnostic Microbiology & Infectious Disease | Study design |
| Nouvenne 2014 (25335100) | Comorbidities and disease severity as risk factors for carbapenem-resistant *Klebsiella pneumoniae* colonization: report of an experience in an internal medicine unit | PloS ONE | Population |
| Orsi 2015  (25411769) | Changed epidemiology of ICU acquired bloodstream infections over 12 years in an Italian teaching hospital | Minerva Anestesiologica | Population |
| Orsi 2006  (16839643) | Hospital-acquired infection surveillance in a neurosurgical intensive care unit | Journal of Hospital Infection | Time of publication |
| Otter 2017  (27746394) | Counting the cost of an outbreak of carbapenemase-producing Enterobacteriaceae: an economic evaluation from a hospital perspective | Clinical Microbiology and Infection | Population |
| Pagano 2014  (No PMID) | Bloodstream infection caused by carbapenemase-producing *Klebsiella pneumoniae* in patients with hematological malignancies | Haematologica | Abstract |
| Palacios-Baena 2016 (26546855) | Comprehensive clinical and epidemiological assessment of olonization and infection due to carbapenemase-producing Enterobacteriaceae in Spain | Journal of Infection | Population |
| Palavutitotai 2018 (29470531) | Epidemiology and risk factors of extensively drug-resistant *Pseudomonas aeruginosa* infections | PloS ONE | Region |
| Pang 2018  (29571291) | Factors associated to prevalence and treatment of carbapenem-resistant Enterobacteriaceae infections: a seven years retrospective study in three tertiary care hospitals | Annals of Clinical Microbiology & Antimicrobials | Region |
| Pang 2018  (No PMID) | Epidemiology of carbapenemase-producing Enterobacteriaceae bacteremia and evaluation of antimicrobial prescribing practices in a community hospital setting | Canadian Journal of Hospital Pharmacy | Abstract |
| Papadimitriou-Olivgeris 2014 (25017796) | Risk factors for infection and predictors of mortality among patients with KPC-producing *Klebsiella pneumoniae* bloodstream infections in the intensive care unit | Scandinavian Journal of Infectious Diseases | Region |
| Peralta 2007 (17644532) | Impact of antibiotic resistance and of adequate empirical antibiotic treatment in the prognosis of patients with *Escherichia coli* bacteraemia | Journal of Antimicrobial Chemotherapy | Time of publication |
| Pintado 2008 (18280570) | Intravenous colistin sulphomethate sodium for therapy of infections due to multidrug-resistant gram-negative bacteria | Journal of Infection | Time of publication |
| Pontikis 2014 (24183799) | Outcomes of critically ill intensive care unit patients treated with fosfomycin for infections due to pandrug-resistant and extensively drug-resistant carbapenemase-producing Gram-negative bacteria | International Journal of Antimicrobial Agents | Region |
| Raviv 2012 (22882693) | Multidrug-resistant *Klebsiella pneumoniae* acquisition in lung transplant recipients | Clinical Transplantation | Region |
| Recio 2018 (29621591) | Bacteraemia due to extensively drug-resistant *Pseudomonas aeruginosa* sequence type 235 high-risk clone: Facing the perfect storm | International Journal of Antimicrobial Agents | Population |
| Rihani 2011 (22200121) | Over-treatment of carbapenemase-producing Enterobacteriaceae | Scandinavian Journal of Infectious Diseases | Study design |
| Rock 2017  (28693656) | Resolution of carbapenemase-producing *Klebsiella pneumoniae* outbreak in a tertiary cancer center; the role of active surveillance | Infection Control & Hospital Epidemiology | Study design |
| Rodrigues Dos Santos 2016 (27569943) | Urinary tract infections and surgical site infections due to carbapenem-resistant Enterobacteriaceae in renal transplant | Transplantation Proceedings | Region |
| Rubio Lopez 2017  (No PMID) | Carbapenemase-producing Enterobacteriaceae in the intensive care unit | Intensive Care Medicine Experimental | Abstract |
| Sacha 2017  (No PMID) | Retrospective evaluation of the use of ceftolozane/tazobactam at a large academic medical center | Infectious Diseases in Clinical Practice | Population |
| Sanchez-Romero 2012 (22005997) | Nosocomial Outbreak of VIM-1-Producing *Klebsiella pneumoniae* Isolates of Multilocus Sequence Type 15: Molecular Basis, Clinical Risk Factors, and Outcome | Antimicrobial Agents and Chemotherapy | Population |
| Sandiumenge 2011 (21659436) | Effect of antibiotic diversity on ventilator-associated pneumonia caused by ESKAPE organisms | Chest | Population |
| Satlin 2017 (28167547) | Multicenter clinical and molecular epidemiological analysis of bacteremia due to carbapenem-resistant Enterobacteriaceae (CRE) in the CRE epicenter of the United States | Antimicrobial Agents & Chemotherapy | Population |
| Satlin 2016 (27404978) | Bacteremia due to carbapenem-resistant Enterobacteriaceae in neutropenic patients with hematologic malignancies | Journal of Infection | Population |
| Satlin 2017 (28167547) | Multicenter clinical and molecular epidemiological analysis of bacteremia due to carbapenem-resistant Enterobacteriaceae (CRE) in the CRE epicenter of the United States | Antimicrobial Agents and Chemotherapy | Duplicate |
| Scheich 2018 (29974230) | Bloodstream infections with gram-negative bacteria and the impact of multidrug resistance in patients with hematological malignancies | Oncology Research and Treatment | Population |
| Sebastien 2018  (No PMID) | Impact of resistant ICU-acquired Gram negative bacilli bloodstream infection on patient prognosis-analysis of a large French ICU network | Annals of Intensive Care | Abstract |
| Sharma 2015  (No PMID) | The outcome of carbapenemase-producing Enterobacteriaceae (CPE) infection in adult solid organ transplant population at a university teaching hospital | American Journal of Transplantation | Abstract |
| Shi 2009  (19638006) | Multidrug resistant gram-negative bacilli as predominant bacteremic pathogens in liver transplant recipients | Transplant Infectious Disease | Time of publication |
| Shields 2016 (26926642) | Aminoglycosides for treatment of bacteremia due to carbapenem-resistant *Klebsiella pneumoniae* | Antimicrobial Agents & Chemotherapy | Study design |
| Shields 2017 (28559250) | Ceftazidime-avibactam Is superior to other treatment regimens against carbapenem-resistant *Klebsiella pneumoniae* bacteremia | Antimicrobial Agents & Chemotherapy | Study design |
| Simonetti 2016  (No PMID) | Oral gentamicin therapy for carbapenem-resistant *Klebsiella pneumoniae* infections in hematologic patients: A single center experience | Haematologica | Abstract |
| Snitkin 2012 (22914622) | Tracking a hospital outbreak of carbapenem-resistant *Klebsiella pneumoniae* with whole-genome sequencing | Science Translational Medicine | Outcome |
| Surgers 2017 (28181033) | Clinical and microbiological determinants of severe and fatal outcomes in patients infected with Enterobacteriaceae producing extended-spectrum beta-lactamase | European Journal of Clinical Microbiology & Infectious Diseases | Population |
| Tölle 2018  (No PMID) | Multidrug resistant gram-negative bacterial blood stream infections in cancer patients-a two years single-center retrospective analysis | Oncology Research and Treatment | Abstract |
| Temkin 2017 (27895014) | Ceftazidime-avibactam as salvage therapy for infections caused by carbapenem-resistant organisms | Antimicrobial Agents & Chemotherapy | Population |
| Tiepke 2017  (No PMID) | Epidemiology, risk factors, and clinical outcome of bloodstream infections caused by gram-negative bacteria in 2015 in a tertiary referral cancer center | Oncology Research and Treatment | Abstract |
| Tofas 2017 (28529091) | *Pseudomonas aeruginosa* bacteraemia in patients with hematologic malignancies: risk factors, treatment and outcome | Diagnostic Microbiology & Infectious Disease | Region |
| Trecarichi 2015 (25595706) | Current epidemiology and antimicrobial resistance data for bacterial bloodstream infections in patients with hematologic malignancies: an Italian multicentre prospective survey | Clinical Microbiology & Infection | Outcome |
| Tsitsopoulos 2016 (27452903) | Nosocomial bloodstream infections in neurosurgery: a 10-year analysis in a center with high antimicrobial drug-resistance prevalence | Acta Neurochirurgica | Region |
| Tuon 2012  (22846123) | Risk factors for pan-resistant *Pseudomonas aeruginosa* bacteremia and the adequacy of antibiotic therapy | Brazilian Journal of Infectious Diseases | Region |
| Tuon 2017  (27821248) | Risk factors for mortality in patients with ventilator-associated pneumonia caused by carbapenem-resistant Enterobacteriaceae | Brazilian Journal of Infectious Diseases | Region |
| Tuon 2015  (25722130) | KPC-producing *Enterobacter aerogenes* infection | Brazilian Journal of Infectious Diseases | Region |
| Vardakas 2015 (25447713) | Characteristics, risk factors and outcomes of carbapenem-resistant *Klebsiella pneumoniae* infections in the intensive care unit | Journal of Infection | Region |
| Vardakas 2015 (25447713) | Characteristics, risk factors and outcomes of carbapenem-resistant *Klebsiella pneumoniae* infections in the intensive care unit | Journal of Infection | Duplicate |
| Vargas-Alzate 2017 (29277527) | High excess costs of infections caused by carbapenem-resistant Gram-negative bacilli in an endemic region | International Journal of Antimicrobial Agents | Region |
| Vergara-Lopez 2015 (25595832) | Lessons from an outbreak of metallo-beta-lactamase-producing *Klebsiella oxytoca* in an intensive care unit: the importance of time at risk and combination therapy | Journal of Hospital Infection | Population |
| Weiner 2016 (26963489) | Vital signs: Preventing antibiotic-resistant infections in hospitals - United States, 2014 | Morbidity & Mortality Weekly Report | Outcome |
| Won 2011  (21865189) | Emergence and rapid regional spread of *Klebsiella pneumoniae* carbapenemase-producing Enterobacteriaceae | Clinical Infectious Diseases | Outcome |
| Wunderink 2018 (30270406) | Effect and safety of meropenem-vaborbactam versus best-available therapy in patients with carbapenem-resistant Enterobacteriaceae infections: The TANGO II randomized clinical trial | Infectious Diseases and Therapy | Population |
| Yuan 2018  (29785131) | Epidemiology, susceptibility, and risk factors for acquisition of MDR/XDR Gram-negative bacteria among kidney transplant recipients with urinary tract infections | Infection and Drug Resistance | Region |
| Zarkotou 2011 (21595793) | Predictors of mortality in patients with bloodstream infections caused by KPC-producing *Klebsiella pneumoniae* and impact of appropriate antimicrobial treatment | Clinical Microbiology & Infection | Region |
| Zhong 2016 (27569958) | *Pseudomonas aeruginosa* infection among liver transplant recipients: A clinical analysis of 15 cases | Transplantation Proceedings | Region |
| Zilberberg 2017 (29225798) | 30-day readmission, antibiotics costs and costs of delay to adequate treatment of Enterobacteriaceae UTI, pneumonia, and sepsis: a retrospective cohort study | Antimicrobial Resistance and Infection Control | Population |
| Zilberberg 2013 (23917908) | Secular trends in gram-negative resistance among urinary tract infection hospitalizations in the United States, 2000-2009 | Infection Control & Hospital Epidemiology | Study design |

**Additional Table 4.** Comparators by outcome for each study

| **Author, year (PMID)** | **Comparator** | **Mortality** | **LOS** | **Cost** | **Readmission** | **Adverse event** | **Mechanical ventilation** |
| --- | --- | --- | --- | --- | --- | --- | --- |
| Alicino 2015 (26464061) | Yes | CSKP |  |  |  |  |  |
| Brizendine 2015 (25385105) | Yes | CSKP |  |  |  |  |  |
| Capone 2013 (23137235) | No | None |  |  |  |  |  |
| Clancy 2013 (24011185) | No | None |  |  |  | None |  |
| Cristina 2018 (28668656) | No | None |  |  |  |  |  |
| Giannella 2018 (28842283) | No | None |  |  |  |  |  |
| Gomez-Simmonds 2015 (25878348) | Yes | CSKP |  |  |  |  |  |
| Hauck 2016 (26850824) | Yes | No infection (colonized) | No infection (colonized) |  |  |  |  |
| Hoxha 2016 (26319590) | Yes | CSKP |  |  |  |  |  |
| Judd 2016 (27320901) | Yes | CSPA | CSPA | CSPA |  |  |  |
| Kalpoe 2012 (22467548) | Yes/no | No CRKP (1-year mortality); None  (30-day mortality) |  |  |  |  |  |
| Mazza 2017 (28457370) | Yes | No CRKP; No infection | No CRKP;  No infection |  |  | No CRKP | No CRKP |
| Messina 2016 (26686227) | Yes |  |  |  | No readmission (patients who weren’t readmitted for CRKP) |  |  |
| Micozzi 2017 (28283020) | No | None |  |  |  |  |  |
| Nguyen 2010 (20356699) | No | None |  |  |  |  |  |
| Pena 2012 (22155832) | Yes | CSPA |  |  |  |  |  |
| Pereira 2015 (26136397) | Yes | CSKP; No infection (colonized) |  |  |  |  |  |
| Pouch 2015 (26341757) | Yes | CSKP |  |  |  | CSKP |  |
| Qureshi 2014 (24637691) | No | None | None |  | None |  |  |
| Salsano 2016 (27371609) | Yes/no | No CRKP (180-day); None  (30-day) | No CRKP |  |  |  |  |
| Satlin 2013 (22916826) | No | None |  |  |  |  | None |
| Simkins 2014 (25092500) | Yes | CSKP |  |  |  | CSKP |  |
| Sotgiu 2018 (29621600) | No | None |  |  |  |  |  |
| Tamma 2016 (28013264) | No | None |  |  |  |  |  |
| Trecarichi 2016 (27428072) | Yes | CSKP |  |  |  |  |  |
| Varotti 2017 (28796391) | Yes | None | No CRKP |  | No CRKP | No CRKP |  |

AE: Adverse event; CR: Carbapenem-resistant; CRKP: Carbapenem-resistant *Klebsiella pneumoniae*; CSKP: Carbapenem-susceptible *Klebsiella pneumoniae*; CSPA: Carbapenem-susceptible *P. aeruginosa*; LOS: Length of stay; KP: *Klebsiella pneumoniae*.

**Additional Table 5.** List of confounders in studies reporting adjusted multivariable analyses

| **Author, year (PMID)** | **Confounders** |
| --- | --- |
| Gomez-Simmonds 2015 (25878348) | Age, Charlson score, Pitt score, ICU admission |
| Hauck 2016 (26850824) | Race, Charlson score |
| Judd 2016 (27320901) | Age, ICU admission |
| Kalpoe 2012 (22467548) | MELD >30 |
| Pereira 2015 (26136397) | Bile leak |
| Pouch 2015 (26341757) | Rejection, receipt of another solid organ at the time of kidney transplant |
| Salsano 2016 (27371609) | NR |
| Trecarichi 2016 (27428072) | Septic shock, acute respiratory failure, initial inadequate antimicrobial therapy |

ICU: Intensive care unit; MELD: Model for End-Stage Liver Disease; NR: Not reported.

**Additional Table 6.** Risk of bias

| **Author, year (PMID)** | **Selection bias: Is the source population (cases, controls, cohorts) appropriate and representative of the population of interest?** | **Performance bias: Is the sample size adequate and is there sufficient power to detect a meaningful difference in the outcome of interest?** | **Detection bias: Did the study use appropriate statistical analysis methods relative to the outcome of interest?** | **Detection bias: Missing data handling was addressed clearly** | **Information bias: Is the methodology of the outcome measurement explicitly stated and is it appropriate?** | **Was the outcome measure objectively (lab measurement)** | **Overall risk** |
| --- | --- | --- | --- | --- | --- | --- | --- |
| Alicino 2015 (26464061) | Low | High | High | Unclear | Low | Unclear | High |
| Brizendine 2015 (25385105) | Low | Low | Low | Unclear | Low | Low | Low |
| Capone 2013 (23137235) | Low | Unclear | Low | Unclear | Low | Low | Unclear |
| Clancy 2013 (24011185) | Low | Unclear | Low | Unclear | Low | Low | Unclear |
| Cristina 2018 (28668656) | Low | Low | High | Unclear | Low | Low | Unclear |
| Giannella 2018 (28842283) | Low | Low | Low | Unclear | Low | Low | Low |
| Gomez-Simmonds 2015 (25878348) | Low | Low | High | Unclear | Low | Low | Unclear |
| Hauck 2016 (26850824) | Low | Low | Low | Unclear | Low | Low | Low |
| Hoxha 2016 (26319590) | Unclear | Low | Low | Unclear | Low | Low | Unclear |
| Judd 2016 (27320901) | Low | Low | Low | Unclear | Low | Low | Low |
| Kalpoe 2012 (22467548) | Unclear | Low | Low | Unclear | Low | Low | Unclear |
| Mazza 2017 (28457370) | Low | Low | Low | Unclear | Low | Low | Low |
| Messina 2016 (26686227) | Low | Low | Low | Unclear | Low | Low | Low |
| Micozzi 2017 (28283020) | Low | Unclear | Low | Unclear | Low | Low | Unclear |
| Nguyen 2010 (20356699) | Low | Low | Low | Unclear | Low | Low | Low |
| Pena 2012 (22155832) | Low | Low | Low | Unclear | Low | Low | Low |
| Pereira 2015 (26136397) | Low | Low | Low | Unclear | Low | Low | Low |
| Pouch 2015 (26341757) | Low | Low | Low | Unclear | Low | Low | Low |
| Qureshi 2014 (24637691) | Low | Low | Low | Low | Low | Low | Low |
| Salsano 2016 (27371609) | Low | Low | Low | Unclear | Low | Low | Low |
| Satlin 2013 (22916826) | Low | Unclear | Low | Unclear | Low | Low | Unclear |
| Simkins 2014 (25092500) | Low | High | Low | Unclear | Low | Low | Unclear |
| Sotgiu 2018 (29621600) | Low | Low | Low | Unclear | Low | Low | Low |
| Tamma 2016 (28013264) | Low | Low | Low | Unclear | Low | Low | Low |
| Trecarichi 2016 (27428072) | Low | Low | Low | Unclear | Low | Low | Low |
| Varotti 2017(28796391) | Low | Low | Low | Unclear | Low | Low | Low |
